# Fruit From Two Kiwifruit Genotypes With Contrasting Softening Rates Show Differences in the Xyloglucan and Pectin Domains of the Cell Wall

**DOI:** 10.3389/fpls.2020.00964

**Published:** 2020-07-02

**Authors:** Christina G. Fullerton, Roneel Prakash, Annu Smitha Ninan, Ross G. Atkinson, Robert J. Schaffer, Ian C. Hallett, Roswitha Schröder

**Affiliations:** ^1^ The New Zealand Institute For Plant & Food Research Limited (Plant & Food Research), Auckland, New Zealand; ^2^ Joint Graduate School of Plant and Food Science, University of Auckland, Auckland, New Zealand

**Keywords:** *Actinidia*, cell wall, enzymes, gene expression, immunolabeling, sugar composition

## Abstract

Fruit softening is controlled by hormonal and developmental cues, causing an upregulation of cell wall-associated enzymes that break down the complex sugar matrices in the cell wall. The regulation of this process is complex, with different genotypes demonstrating quite different softening patterns, even when they are closely related. Currently, little is known about the relationship between cell wall structure and the rate of fruit softening. To address this question, the softening of two *Actinidia chinensis* var. *chinensis* (kiwifruit) genotypes (a fast ‘AC-F’ and a slow ‘AC-S’ softening genotype) was examined using a range of compositional, biochemical, structural, and molecular techniques. Throughout softening, the cell wall structure of the two genotypes was fundamentally different at identical firmness stages. In the hemicellulose domain, xyloglucanase enzyme activity was higher in ‘AC-F’ at the firm unripe stage, a finding supported by differential expression of xyloglucan transglycosylase/hydrolase genes during softening. In the pectin domain, differences in pectin solubilization and location of methyl-esterified homogalacturonan in the cell wall between ‘AC-S’ and ‘AC-F’ were shown. Side chain analyses and molecular weight elution profiles of polyuronides and xyloglucans of cell wall extracts revealed fundamental differences between the genotypes, pointing towards a weakening of the structural integrity of cell walls in the fast softening ‘AC-F’ genotype even at the firm, unripe stage. As a consequence, the polysaccharides in the cell walls of ‘AC-F’ may be easier to access and hence more susceptible to enzymatic degradation than in ‘AC-S’, resulting in faster softening. Together these results suggest that the different rates of softening between ‘AC-F’ and ‘AC-S’ are not due to changes in enzyme activities alone, but that fundamental differences in the cell wall structure are likely to influence the rates of softening through differential modification and accessibility of specific cell wall polysaccharides during ripening.

## Highlights

Different softening rates in kiwifruit are the result of differential enzyme activities, cell wall structure, and accessibility of specific cell wall polysaccharides during ripening.

## Introduction

Fruit ripening is the final step in fleshy fruit development, with softening of the flesh being a key part of the process. Fruit softening is initiated and sustained by developmental and hormonal signals, and at the biochemical level it involves extensive remodeling and breakdown of the cell wall structure (Wang et al., 2018). The major components of fruit cell walls are pectins, hemicelluloses, and cellulose. Pectin consists mainly of subpopulations of homogalacturonan (HG) and rhamnogalacturonan I (RG-I). HGs are linear chains of galacturonic acid, whereas RG-I is a heteropolymer composed of alternating rhamnose and galacturonic acid residues. Attached to the rhamnose residues are galactan and arabinan side chains. HG and RG-I comprise the backbone of pectin, and this backbone and the RG-I side chains can be arranged in any pattern and in any length (Mohnen, 2008). Xyloglucan is the main hemicellulose and is composed of a *β*-(1→4) glucan backbone substituted with short side stubs of xylose, galactose, and sometimes fucose. A strong network forms through xyloglucan hydrogen-bonding to the surface of cellulose microfibrils, thereby cross-linking them (Pauly et al., 1999; Cosgrove, 2005). There is also evidence for strong covalent interactions between pectin populations and cellulose (Cavalier et al., 2008; Höfte et al., 2012; Park and Cosgrove, 2012; Broxterman and Schols, 2018) with galactans additionally acting as anchors (Zykwinska et al., 2005; Zykwinska et al., 2007). This suggests pectins may play more of a role in maintaining the structural integrity of the cell wall than previously thought.

Most changes during softening involve pectin and xyloglucan, influencing their interactions with cellulose and thereby weakening the cell wall. These changes include solubilization of pectin, removal of arabinose and galactose from galactan and arabinan side chains of RG-I pectin, demethylesterification of HG, and depolymerization of pectin and hemicelluloses. These processes are common features of cell wall changes in many fleshy fruits during softening, although they may vary regarding timing and extent between species (Toivonen and Brummell, 2008). Pectin degradation occurs as the result of coordination and interaction among enzymes including pectin methylesterase (PME), polygalacturonase (PG), *β*-galactosidase (BGal), and *α*-L-arabinofuranosidase (AFase). PME removes methyl groups of HG, while PG cleaves the galacturonic linkages of the HG backbone (Brummell, 2006). Transgenic studies on apple and strawberry have indicated a central role of PG in fruit softening, as downregulation of PG in these species leads to a slower softening rate compared to the wild type (Quesada et al., 2009; Atkinson et al., 2012; Posé et al., 2013). BGal and AFase are characterized by their ability to cleave galactose residues of galactan and arabinan RG-I sidechains, respectively. In tomato, a family of BGal enzymes is collectively responsible for the removal of galactose residues from galactan side chains (Carey et al., 2001). In transgenic studies using tomato, downregulation of tomato BGal gene *TBG4* resulted in a firmer fruit (Smith et al., 2002; Liu et al., 2018), whereas downregulation of *TBG1* had little effect (Carey et al., 2001). Additional evidence showed that downregulation of BGal genes in peach may also decrease PG and PME transcription and activity, suggesting an interconnection between cell wall enzyme activities. AFase removes the terminal nonreducing arabinofuranosyl residues from *α*-(1→5)-arabinan side chains of RG-I. Increased *α*-L-arabinofuranosidase activity during ripening or storage was observed in apple, Japanese pear fruit, avocado, tomato, persimmon, peach, and European and Chinese pear (reviewed in Tateishi, 2008) and has been associated with the development of mealiness in apple (Nara et al., 2001) and peach (Brummell et al., 2004).

The main xyloglucan-modifying enzyme is xyloglucan endotransglycosylase/hydrolase (XTH) which potentially possesses two enzymatic activities: xyloglucanase and xyloglucan endotransglycosylase (XET) activity. Most XTH enzymes act mainly as XETs and have no xyloglucanase activity, whereas only a few act predominantly as xyloglucanases (Rose et al., 2002). While true dual activity of XTH enzymes are rare, Schröder et al. (1998) showed that the gene product of *AdXET6* (which corresponds to XTH6 in this current study) purified from the core tissue of *Actinidia chinensis* var. *deliciosa* ‘Hayward’ showed both XET and xyloglucanase activity *in vitro*, depending on the nature of the substrates available. XTH has been shown to be a wall-loosening enzyme and may play an important role in cell wall turnover and maintenance (Hiwasa et al., 2004; Lu et al., 2006; Nishiyama et al., 2007). XTH has also been related with fruit softening, particularly in fruit where xyloglucan depolymerization occurs such as in persimmon, kiwifruit, and tomato (Cutillas-Iturralde et al., 1994; Maclachlan and Brady, 1994). XET activity has been shown to increase in kiwifruit during softening, especially in the core tissue (Redgwell and Fry, 1993). In kiwifruit and tomato, the increase in XET activity was accompanied by a reduction in the relative molecular mass of xyloglucan (Redgwell and Fry, 1993; Maclachlan and Brady, 1994).

Expansins (EXPs) are a family of non-enzymic proteins that have been proposed to weaken the hydrogen bonds between cellulose and xyloglucan, temporarily loosening the cell wall (McQueen-Mason and Cosgrove, 1994). While increases in *EXP* in fruit softening have been well described (Cosgrove, 2000; Brummell and Harpster, 2001), their function in this process is not entirely understood. Brummell et al. (1999) showed that downregulation of a tomato expansin *LeExp1* inhibited pectin depolymerization late in ripening but had no influence on the breakdown of hemicelluloses, whereas overexpression of this gene resulted in softer fruit compared to controls, also with extensive depolymerization of hemicelluloses but with no alteration in pectin depolymerization.

The phytohormone ethylene is a critical regulator of climacteric fruit softening (Lelièvre et al., 1997; Vishwas et al., 2010). Climacteric and nonclimacteric fruit are distinguished by the presence or absence of the rise in respiration (climacteric) coinciding with autocatalytic ethylene production (Inaba, 2007). Various cell wall-modifying enzymes, such as BGal and PME, have been shown to increase in expression in response to ethylene during ripening of different fruits including tomato, strawberry, melon, apple, peach and kiwifruit (Trainotti et al., 2001; Alexander and Grierson, 2002; Castillejo et al., 2004; Cara and Giovannoni, 2008; Pech et al., 2008; Muñoz-Bertomeu et al., 2013). Tomato is the predominant model for studying climacteric fruit ripening, and the control of ethylene through epigenetic and transcriptional regulation has been well documented (Giovannoni et al., 2017), with key regulators such as Ripening Inhibitor (Rin) and Non Ripening (Nor) controlling ethylene production (Giovannoni et al., 2017; Lü et al., 2018).

Kiwifruit is an unusual climacteric fruit as many ripening processes, including the majority of fruit softening, occur independently of climacteric ethylene (Richardson et al., 2011). Climacteric (endogenous) ethylene production starts at the last stage of kiwifruit ripening (Atkinson and Schroder, 2016), when fruit is becoming eating-soft, but can also be induced by exogenous ethylene or chilling exposure (Minas et al., 2016). When ethylene is suppressed, the last stages of ripening including the production of aroma volatiles do not occur (Atkinson et al., 2011). These studies have shown that, although classified as a climacteric fruit, the softening behavior differs from the tomato ripening model, and it has been proposed that this may be due to a difference in the way that a *RIN*-like gene is regulated (McAtee et al., 2015). Commercial kiwifruit rarely produce endogenous ethylene when unripe at harvest, however during fruit ripening they are extremely sensitive to exogenous ethylene (McAtee et al., 2015). This suggests that although ethylene is the main regulator in climacteric fruit, both ethylene-dependent and ethylene-interdependent gene regulation pathways must coexist to co-ordinate the ripening process (Lelièvre et al., 1997).

Fruit physiology is important when understanding how kiwifruit may behave postharvest. Of particular importance are kiwifruit maturity (Burdon, 2018) and softening behavior, investigated using kiwifruit cultivars *Actinidia chinensis* var. *deliciosa* ‘Hayward’ (Schröder and Atkinson, 2006; Burdon et al., 2017) and *Actinidia chinensis* var. *chinensis* ‘Hort16A’ (Gunaseelan et al., 2019). Different kiwifruit genotypes can exhibit variable rates of softening, even when very closely related (White et al., 2005). However, currently little is known about the relationship between cell wall structure and the rate of fruit softening. To investigate this further, two noncommercial kiwifruit genotypes with a similar genetic background were investigated, a fast softening *Actinidia chinensis* var. *chinensis* genotype ‘AC-F’, and a slow softening *A. chinensis* var. *chinensis* genotype ‘AC-S’. Our aim was to determine whether the contrasting softening rates of these two fruits are a result of a) fundamental differences in the cell wall chemistry resulting in a structure that changed the way the enzymes could degrade the wall, or b) alterations in the rate of gene expression and activity of cell wall enzymes resulting in slower or faster modifications of the cell walls.

## Material and Methods

### Physiological Assessments of Fruit and Tissue Sampling

*Actinidia chinensis* Planch. var. *chinensis* ‘AC-S’ is a slow softening, large fruited, advanced breeding selection. ‘AC-F’ is a fast softening genotype and the female parent of a large interspecific population used in previous physiological studies (*e.g.* Nieuwenhuizen et al., 2012). Both genotypes were grown at the Plant & Food Research orchard in Te Puke. The fruit was harvested in April 2012 (‘season 1’) and 2013 (‘season 2’) at similar physiological maturity based on the harvest index developed for ‘Hayward’ in New Zealand (movement from 6.2% Brix) (Harman, 1981). Methods for fruit sampling were established in ‘season 1’ and repeated in replicated trials in ‘season 2’.

The fruit was ripened at 20°C in trays containing 30 pieces of fruit, each tray covered with breathable polyliners. The ripening and softening progress was monitored by determining firmness, soluble solid concentration, dry matter (DM), and ethylene production of the fruit (Fullerton, 2015), using 30 pieces of fruit per phenotyping day. DM was measured just once, at harvest, as it does not change during the period of kiwifruit harvest (Burdon et al., 2016). ‘AC-F’ was phenotyped daily during softening, and ‘AC-S’ every 2–4 days after checking by touch if the fruit had progressed in softening. During softening and phenotyping, the fruit was sampled into four firmness categories (FCs) according to their outer pericarp firmness. These four stages represented the four typical phases of kiwifruit ripening reported by Schröder and Atkinson (2006). The fruit of FC1 was unripe and very firm (70–80 N); FC2 had started softening but were still firm and unripe (40–60 N); FC3 approached the edible phase (10–30 N); and the fruit of FC4 was at an edible firmness and ripe (< 7 N). For the fast softening genotype, FC2 and FC3 were created by sampling subpopulations of appropriate firmness within day 2.

In ‘season 1’ and ‘season 2’, outer pericarp tissue from at least ten pieces of fruit at each of these four firmness stages was pooled, diced and snap frozen in liquid nitrogen, and stored at −80°C for cell wall-, enzyme-, and expression analyses.

### Light Microscopy and Immunolabeling

**Figure 1:**
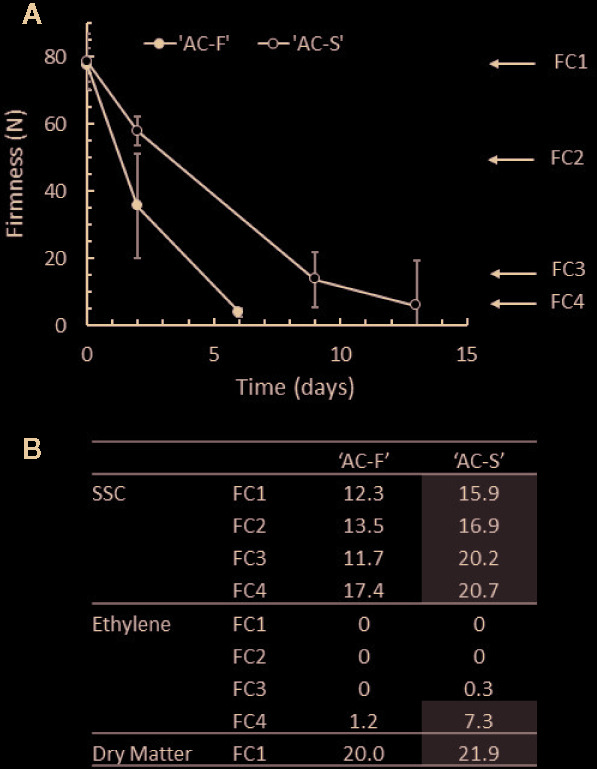
Ripening characteristics of *Actinidia chinensis* var. *chinensis* genotypes ‘AC-F’ (fast softening) and ‘AC-S’ (slow softening) during ripening at 20°C in ‘season 2’. **(A)** Change in the firmness of the outer pericarp of ‘AC-F’ and ‘AC-S’ during softening. **(B)** Soluble solid concentration (SSC) in °Brix, endogenous ethylene production in nmol ethylene·kg fruit^−1^·s^−1^ and dry matter in % throughout softening. Values are means ± standard deviation (n = 30) of ‘season 2’ fruit. The ripening characteristics of season 1 are summarized in **Supplementary Table S4**.

Preparation of samples for microscopy and localization of cell wall epitopes was carried out according to Sutherland et al. (2009) and Ng et al. (2013). In brief, blocks of outer pericarp tissue were excised from three replicates of the fruit at softening stages FC1 and FC4 (**Figure 1A**), fixed in 2% paraformaldehyde/glutaraldehyde, dehydrated in an ethanol series, and embedded in LR White resin (London Resin, UK). Embedded material was trimmed and sections were cut at 1 µm (toluidine blue) or 200 nm (immunolabeling), mounted on poly-L-Lysine coated slides, and dried overnight at 45°C. Each slide held three sections from each fruit replicate per firmness stage.

For bright field observation, tissue sections (1 μm) were stained with 0.05% toluidine blue in benzoate buffer pH 4.4, washed with water, and air-dried. Sections were mounted in SHUR/Mount™ (Triangle Biomedical Sciences, USA) and examined using transmitted light on an Olympus Vanox AHBT3 compound microscope (Olympus Optical Co. Ltd., Japan). Images were captured using a Roper Scientific CoolSnap camera (Roper Scientific Ltd., USA), and representative images were chosen for illustration.

Immunolabeling was carried out on 200 nm sections using the monoclonal antibodies JIM5, JIM7, LM5 and LM15 (PlantProbes, UK) (**Supplementary Table S1**) as described in Sutherland et al. (2009). Each slide was encircled with a hydrophobic barrier using a PAP pen (Daido Sangyo Co. Ltd., Japan). After air-drying, sections were blocked with 0.1% bovine serum albumin (BSA-c, Aurion, Netherlands) in phosphate buffered saline (PBS) plus 0.1% Tween 80, incubated with the monoclonal antibody and washed again. Sections were then incubated with the secondary antibody (goat anti-rat IgG AlexaFluor 488. Invitrogen, USA) diluted 1:600 in PBS; washed and allowed to air dry. Samples were mounted in Citifluor AF1 (Citifluor, UK) and viewed under epifluorescence with the Vanox compound microscope using a mercury light source and an Olympus BH2-DMIB interference blue filter set (excitation 495 nm, longpass emission from 515) collecting images with the CoolSnap camera. All images presented are based on fixed exposure times which allow for differences in labeling intensity as well as pattern of localization to be interpreted. Representative images were chosen for illustration.

### Cell Wall Preparation, Extraction and Analyses

Cell wall isolation and extraction were performed on outer pericarp tissue from the fruit over two seasons, as described in Ng et al. (2013). In brief, after obtaining the water-soluble extract (WS), dimethyl sulfoxide soluble extract (DMSO) and cell wall material (CWM) from 50 g of frozen, finely ground tissue at each firmness stage in two seasons, the CWM was sequentially extracted with 0.05 M trans-1,2-diaminocyclohexane-N,N,N′,N′-tetraacetic acid (CDTA), followed by 0.05 M Na_2_CO_3_ containing 20 mM NaBH_4_, 1 M KOH containing 20 mM NaBH_4_, and 4 M KOH containing 20 mM NaBH_4_. After each extraction, the insoluble pellets were re-suspended, centrifuged, filtered, and rinsed twice with water. Supernatants were combined accordingly to give the CDTA-, Na_2_CO_3_-, 1 M KOH-, and 4 M KOH-soluble extracts. The remaining insoluble pellet was termed cell wall residue (CWR). The soluble extracts as well as CWM and CWR were dialyzed to remove salts (MWCO 10 kDa), freeze-dried and weighed to determine yields. Cell wall material from ‘season 2’ was prepared from three biological replicates and CWM from ‘season 1’ from one biological replicate. Cell walls were sequentially extracted using one biological replicate each from ‘season 1’ and ‘season 2’.

Neutral sugar content was determined by gas chromatography (GC) after hydrolysis in trifluoroacetic acid (TFA), followed by the conversion of monosaccharides to alditol acetates (Albersheim et al., 1967) as described in Prakash et al. (2017). Each neutral sugar analysis was carried out twice per firmness category with two technical replicates per biological replicate. Uronic acids (UAs) were determined as described by Ahmed and Labavitch (1977) and Blumenkrantz and Asboe-Hansen (1973) with galacturonic acid as a standard. Each UA analysis was carried out three times per firmness category with three technical replicates per biological replicate.

Analysis of the pectin side chains of CWM from ‘season 2’ was carried out according to the method of Renard and Ginies (2009), based on molar ratios of UA, rhamnose, galactose and arabinose calculated from neutral sugar and UA analyses. The relative amounts of HG and RG-I regions in the pectin backbone are given as the UA to rhamnose ratio. The ratio of the sum of arabinose and galactose residues to rhamnose is indicative of the relative amounts of galactan and arabinan attached to the RG-I backbone, whereas the ratio of arabinose to galactose provides an estimate of the proportions of arabinan *versus* galactan as side chains of RG-I.

### Size Exclusion Chromatography

Water-, CDTA- and Na_2_CO_3_-soluble extracts were hydrated in water (1 mg · ml^−1^) and eluted through Superose 6 (column 1 × 30 cm; eluent 0.05 M ammonium acetate buffer pH 5.0; flow rate 0.5 ml · min^−1^). Fractions (0.25 ml) were collected and elution profiles created by assaying fractions for UA according to Blumenkrantz and Asboe-Hansen (1973). UA content of each fraction was determined using galacturonic acid as standard and results presented as μg UA· fraction^−1^ · μg^−1^ total UA content loaded.

The 1 M KOH and 4 M KOH soluble extracts (2–3 mg) were dissolved in 1 ml of water and passed through Sephacryl S-300 (column 1.5 × 75 cm; eluent 0.05 M Na acetate, 0.125 M NaCl; flow rate approximately 6 ml · h^−1^. Fractions (20 min) were collected and elution profiles created by assaying fractions for UA and total carbohydrate content. UA was determined as described above. Total carbohydrate content was determined by the phenol-sulfuric assay for microtiter plates (van den Hoogen et al., 1998). Absorbance was measured at 490 nm, and total carbohydrate content was calculated using glucose (0–0.40 mg · ml^−1^) as standard. Results were presented as µg total carbohydrate · fraction^−1^ · µg^−1^ total carbohydrate content loaded.

Column runs were carried out twice per extract at firmness stages FC1 and FC4 for each genotype and extract over two seasons. Representative runs are shown. Columns were calibrated with glucose (180 kDa), dextrans T10 (10 kDa), T40 (40 kDa), T500 (500 kDa), and Blue Dextran (> 2000kDa) (Pharmacia).

### Gene Expression Analysis

Total RNA was extracted from kiwifruit tissue of ‘season 2’ in three biological replicates according to the method of Chang et al. (1993) and treated with DNase (Ambion, Applied Biosciences) according to the manufacturer’s instructions to remove contaminating genomic DNA. RNA concentration was measured using a NanoDrop^®^ ND-2000 UV-Vis Spectrophotometer (NanoDrop Technologies, Thermo Scientific, USA) at 260 nm. First-strand cDNA was synthesized from 500 ng of total RNA (Tetro cDNA synthesis kit, Bioline) according to the manufacturer’s instructions. qPCR reactions were performed in quadruplicate using LightCycler^®^ 480 SYBR Green I Master Mix (Roche Diagnostics) (5 µl), primers (2 µl; 0.5 µM final concentration) and cDNA diluted in nuclease free water (1:40) (3 µl). A negative control was included in each run with nuclease free water used in place of cDNA. The qPCR conditions were 5 min at 95°C followed by 40 cycles of 5 s at 95°C, 5 s at 60°C, and 10 s at 72°C followed by 65–95°C melting curve detection. Primer sequences associated with the new gene models described in Pilkington et al. (2018) are listed in **Supplementary Table S2**. Differences in gene expression were initially tested to find the best housekeeping gene from ACTIN, EF1α and protein phosphatase 2A (*PP2A*). The results presented here are normalized to kiwifruit ACTIN as described in McAtee et al. (2015).

### Enzyme Extractions, Assays and Quantification of Activity

Xyloglucan endotransglycosylase (XET), xyloglucanase, BGal, and PME activity were extracted from frozen tissue as described in Prakash et al. (2017), using a low salt (LS)–high salt (HS) buffer extraction approach. Enzyme activity assays were carried out using at least three extracts derived from bulk tissue (~10 pieces of fruit per softening stage) collected over ‘season 2’, with three to five technical replicates for each assay including appropriate controls.

PME activity was assayed based on proton release based on Guglielmino et al. (1997) as described in Fullerton (2015). PME activity is given as μmol GalA released · min^−1^ · g^−1^ fresh weight.

BGal activity was determined by its ability to cleave p-nitrophenyl-*β*-D-galactopyranoside substrate (Sigma) and enzyme activity reported as mol *p*-nitrophenol released · h^−1^ · g^−1^ fresh weight using a standard curve constructed with *p*-nitrophenol made up in LS or HS extraction buffer as described in Prakash et al. (2017).

XET activity was assayed by its ability to attach tritium-labeled xyloglucan-derived oligosaccharide substrates to xyloglucan polysaccharide substrates after Schröder et al. (1998). XET activity is given as Bq of radioactivity incorporated into high molecular products · kBq of radioactive oligosaccharides supplied^−1^ · fresh weight^−1^ · h^−1^ unless stated otherwise.

Xyloglucanase activity was assayed by gel diffusion as described in Prakash et al. (2017). The radius of the clearance zones after staining of the agar with Congo Red was digitally measured after photographing the plates (Nikon D80 digital SLR camera, with a 60 mm macro Nikkor lens, Nikon Corporation, Japan) using a program written in the programming language MATLAB^®^ which analyzes the image of a plate in RGB (red, green, blue) format. As the contrast between areas of enzyme activity and areas of no enzyme activity was mostly in the green color channel of the image, activity was measured by counting the number of pixels that had green color intensity between defined minimum and maximum values for each radius. Pixels were converted to mU using the standard curves created with known concentrations of commercial XGase (endocellulase from *Trichoderma longibrachiatum* EC 3.2.1.4; Megazyme) in appropriate LS and HS buffers, and results are expressed in mU of enzyme · g^−1^ fresh weight.

### Heterologous XTH Recombinant Proteins: Expression, Purification and Enzymatic Activity Assays

His-tagged recombinant XTH5, XTH7, and XTH13 proteins were expressed in *Escherichia coli* and purified as described in Atkinson et al. (2009). After determination of the protein content of each eluted fraction, protein-containing fractions were tested for XET activity as described in Schröder et al. (1998) using 10 µl per fraction in an overnight assay. XET-positive fractions were pooled, the protein concentration determined, and aliquots tested for XET and xyloglucanase activity. Recombinant enzyme extractions were carried out twice.

XET activity was determined in triplicate from each of the recombinant enzyme extracts as described in Schröder et al. (1998) using 10 µl of pooled XET-positive fractions, 20 µl substrate solution (0.25% tamarind xyloglucan from Megazyme, 283 kBq mL^−1^ [3H]XXXG-ol in water), 10 µl 1 M MES buffer pH 5.8, 20 µl water and incubated overnight at 23°C, followed by the paper assay and quantification of radioactivity bound to paper. XET activity is given as Bq of radioactivity incorporated into high molecular products · kBq^−1^ of radioactive oligosaccharides supplied · mg^−1^ protein.

Xyloglucanase activity was determined by gel diffusion assays in duplicate as described above from each of the recombinant enzyme extracts. Results are given in mU of enzyme · mg^−1^ protein. Recombinant XTHs from both extracts were also incubated for 24 h with tamarind xyloglucan at 23°C (40 µl of a 1% w/v solution, protein concentration 0.693 mg, total volume 2 ml adjusted with 0.2 M MES pH 5.8) and the total mixture subjected to gel filtration chromatography on Superose 6 as described above. Elution was monitored using the phenol-sulfuric assay for microtiter plates (van den Hoogen et al., 1998) and results of a typical run presented as OD _525 nm_ · fraction^−1^.

### Statistical Analyses

Excel 2013 was used to perform two-tailed *t* tests to calculate the significance of sample means (*p* < 0.05) between genotypes in terms of cell wall composition, enzyme activity, and molecular analysis.

## Results

### Ripening Characteristics of Fast Softening ‘AC-F’ and Slow Softening ‘AC-S’ Genotypes

The ripening characteristics of two related kiwifruit genotypes with differential softening characteristics (fast softening ‘AC-F’ and slow softening ‘AC-S’) were analyzed in detail at four defined softening stages during ripening (FC1–FC4) (Schröder and Atkinson, 2006) over two seasons. When kept at 20°C, ‘AC-F’ softened in 6 days from FC1 to FC4, whereas ‘AC-S’ needed 13 days to reach the same softness (‘season 2’, **Figure 1A**). At harvest, the DM content differed only slightly between genotypes being 21.9 *vs.* 20.0% in ‘AC-S’ and ‘AC-F’ respectively. This difference in DM content may account for the differences seen in SSC at FC1 (being 3.6% higher in ‘AC-S’ compared with ‘AC-F’). SSC content increased significantly in both genotypes over softening. At FC4, the SSC was significantly lower in ‘AC-F’ compared with ‘AC-S’ (**Figure 1B**). The increase in SSC is due to conversion of starch into sugar during ripening. The DMSO-soluble extracts that arise while preparing cell walls contain mostly starch, and yields are therefore indicative of starch degradation. Yields were highest in firm, unripe fruit at FC1 in both genotypes and decreased as softening progressed. However, at FC1 and FC2, ‘AC-S’ yielded approximately twice the amount of DMSO-soluble material than ‘AC-F’ (**Supplementary Table S3**). Ethylene production was observed at negligible or very low levels in both genotypes at FC3 and reached its maximum at FC4 with quite wide variability of production in ‘AC-S’ fruit (**Figure 1B**).

Similar changes in ripening characteristics between ‘AC-F’ and ‘AC-S’ were obtained in ‘season 1’ (**Supplementary Table S4**).

### Cell Size and Degree of *In Vivo* Cell Wall Swelling Are Different in ‘AC-F’ and ‘AC-S’

**Figure 2:**
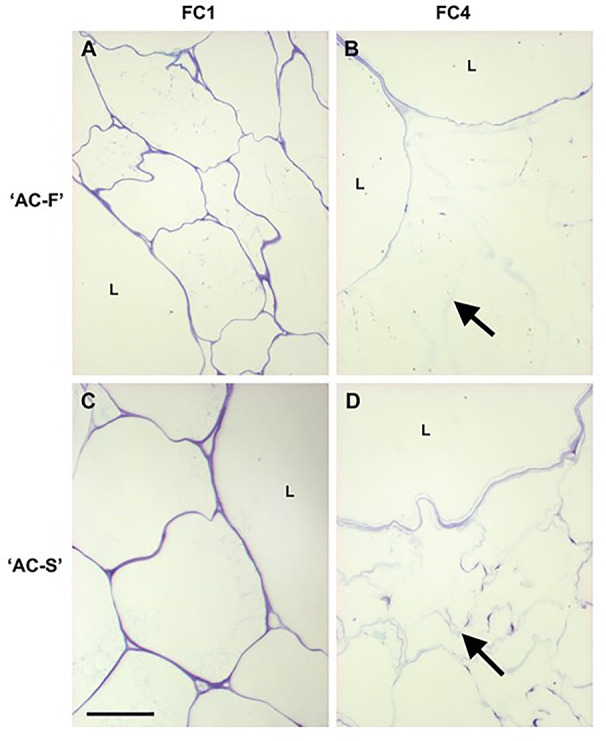
Toluidine blue staining of cell walls of fast softening *Actinidia chinensis* var. *chinensis* ‘AC-F’ and slow softening ‘AC-S’ during ripening. Toluidine blue stained resin-embedded section of ‘AC-F’ **(A, B)** and ‘AC-S’ **(C, D)** at FC1 and FC4. Bar = 50 µm in all images. Arrows indicate blue outline of small cells. L, large cells; FC, firmness category.

The outer pericarp tissue of both ‘AC-F’ and ‘AC-S’ comprised large and small parenchyma cells, which is typical for kiwifruit (**Figure 2**). In cross-section, the large cells of both genotypes were nearly circular and of the same dimensions. The small cells of ‘AC-F’, however, were more irregular in shape and appeared to be half the size of those in ‘AC-S’ (**Figures 2A, C**). Both genotypes showed a reduction in the intensity of toluidine blue staining during softening, indicating loss of cell wall integrity. This was more pronounced in the small cells (outline indicated by arrows in **Figures 2B, D**). The large cells retained a greater degree of cell wall staining than small cells, suggesting that they also retained more of their cell wall integrity (**Figures 2B, D**). Differences in staining and cell wall swelling between genotypes were observed at the ripe FC4 stage. In ‘AC-F’, the large cells were strongly stained and showed no swelling, while the small cells exhibited cell wall swelling and lost most staining ability (**Figure 2B**). In ‘AC-S’, the large cells showed some degree of swelling. Additionally, discrete regions of the cell wall in the small cells showed staining, probably indicating the presence of plasmodesmatal pit fields (**Figure 2D**). This was also seen in ‘AC-F’, although with less intensity (**Figure 2B**).

### Methylesterification Patterns of Homogalacturonan (HG), Galactan and Xyloglucan Distribution Are Associated With Differences in the Softening Rate of ‘AC-F’ and ‘AC-S’

**Figure 3:**
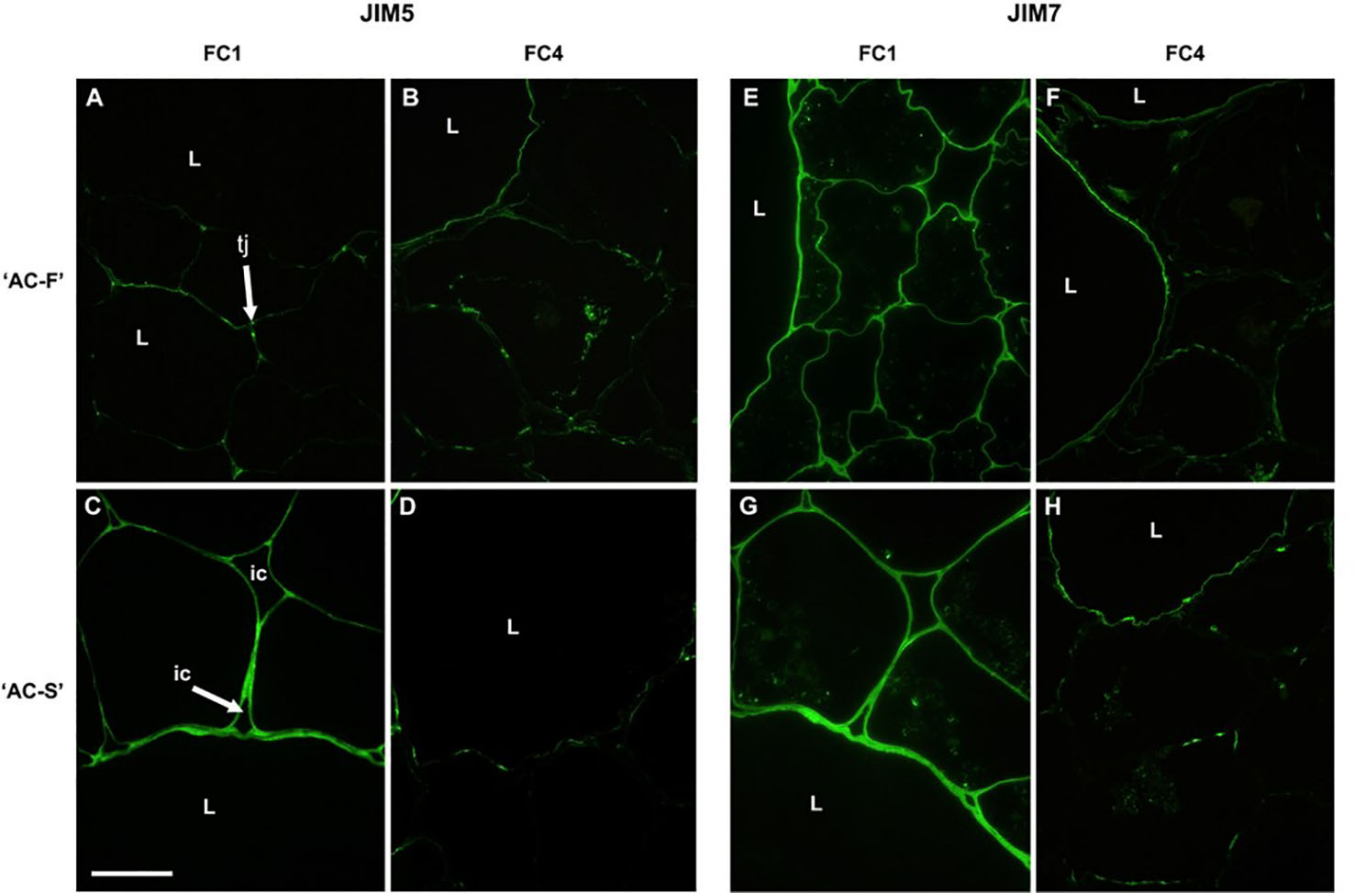
Immunofluorescence labeling with the antibody JIM5 (low methylesterified or unesterified homogalacturonan) **(A–D)** and with JIM7 (highly esterified homogalacturonan) **(E–H)** in the *Actinidia chinensis* var. *chinensis* fast softening genotype ‘AC-F’ **(A, B, E, F)** and slow softening genotype ‘AC-S’ **(C, D, G, H)** at FC1 and FC4. Bar = 50 µm for all images. tj, tricellular junction; ic, intercellular spaces; L, large cells; FC, firmness category.

Monoclonal antibodies JIM5 (recognizing low- or un-esterified HG regions) and JIM7 (recognizing esterified HG regions) revealed different esterification patterns of HG in cell walls of ‘AC-F’ and ‘AC-S’. In FC1, labeling of JIM5 epitopes was concentrated at tricellular junctions between cells or junctions between two cells and an intercellular space in both genotypes. ‘AC-S’ also showed considerable labeling of cell walls of large and small cells (**Figure 3C**), whereas in ‘AC-F’ only random regions were labeled (**Figure 3A**). In ‘AC-F’ fruit at FC4, JIM5 labeling was still visible as continuous lines at the cell–lumen interface of the large cells and in the small cells as randomized patches of labeling at the cell lumen interface and the middle lamella region (**Figure 3B**). Although JIM5 labeling was stronger in ‘AC-S’ at FC1, ripe fruit at FC4 showed only weak patchy labeling located at the cell lumen interface in the cell walls of the large cells. In the small cells, most of the labeling had been lost (**Figure 3D**).

Immunolabeling with JIM7, a monoclonal antibody that labels esterified regions of homogalacturonan, was localized across the cell walls of both large and small cells in both genotypes at FC1; however, labeling appeared more intense in ‘AC-S’ than in ‘AC-F’ (**Figures 3E, G**). Once the fruits were ripe, JIM7 labeling intensity had decreased in both genotypes. In ‘AC-F’, labeling was located in a thin region of the cell wall of large cells, possibly at the lumen face, and had become patchy in the small cells. In ‘AC-S’, labeling was mainly present in the large cells; however the pattern was less uniform. The walls of the small cells were poorly defined and had lost most of their ability to be labeled (**Figures 3F, H**).

**Figure 4:**
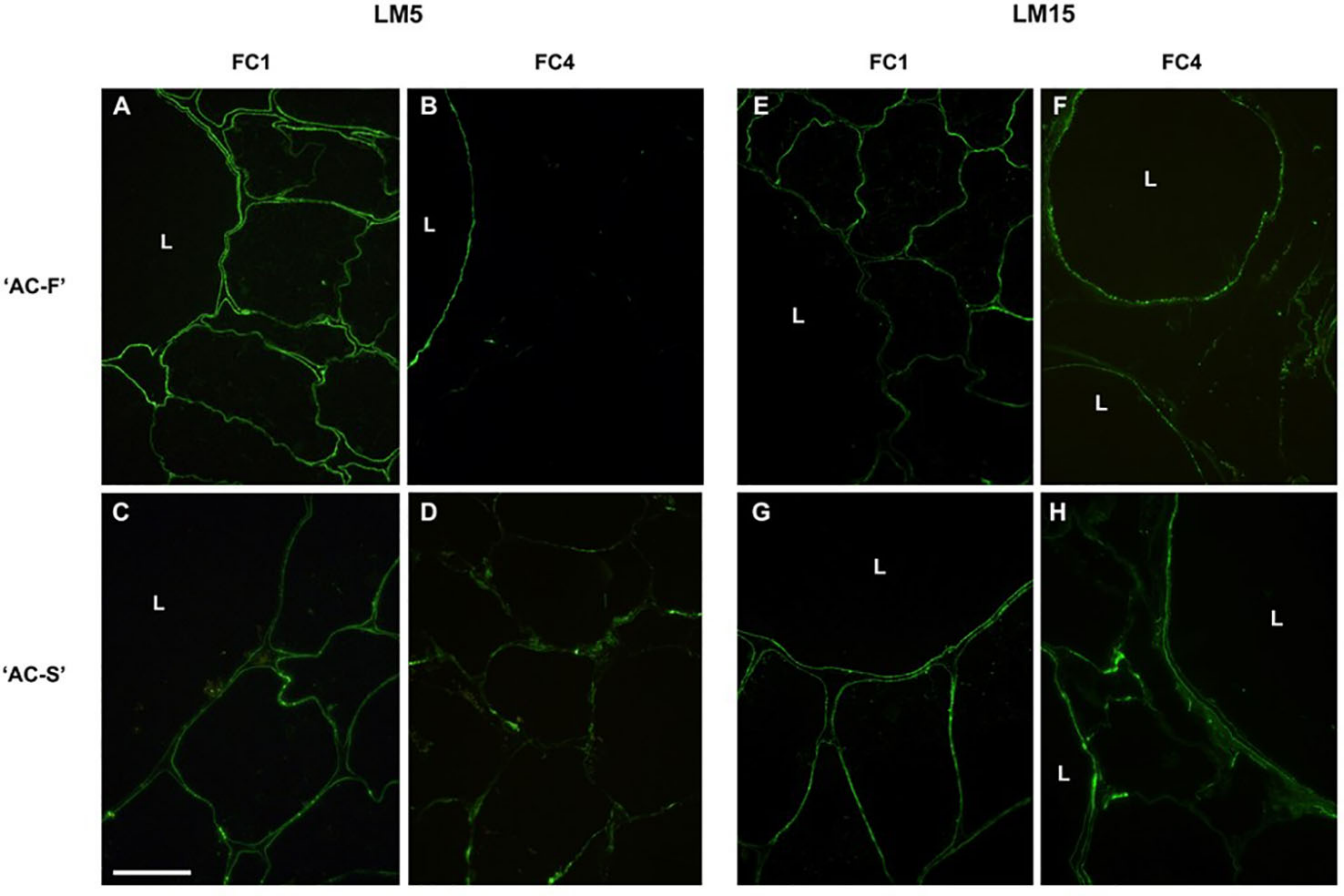
Immunofluorescence labelling of *Actinidia chinensis* var. *chinensis* fast softening genotype ‘AC-F’ **(A**, **B**, **E**, **F)** and slow softening genotype ‘AC-S’ **(C**, **D**, **G**, **H)** with monoclonal antibody LM5 [specific for (1-4)-*β*-D-galactan] and LM15 (specific for the XXXG motif of xyloglucan) at FC1 and FC4. Bar = 50 µm for all images. L, large cells; FC, firmness category.

Immunolabeling with LM5, a monoclonal antibody specific for galactan side chains, revealed only weak labeling in the cell walls of both the large and small cells and was located on the lumen side of the cell walls in both genotypes (**Figures 4A, C**). At FC4, only large cells showed labeling in the‘AC-F’, whereas the small cells in the ‘AC-S’ had retained some epitopes for binding of LM5 (**Figures 4B, D**).

Immunolabeling with LM15 specific for the xyloglucan epitope XXXG showed uneven, speckled labeling at the cell–lumen interface in the cell walls of large and small cells in both genotypes at FC1 (**Figures 4E, G**). At FC4, speckled labeling remained at the cell–lumen interface of the large cells in ‘AC-F’ but had been lost in the small cells. In ‘AC-S’ on the other hand, patchy LM15 labeling was visible in the cell walls of both the large and small cells (**Figures 4F, H**).

### ‘AC-F’ and ‘AC-S’ Genotypes Have Different Cell Wall Sugar Composition Throughout Softening

**Figure 5:**
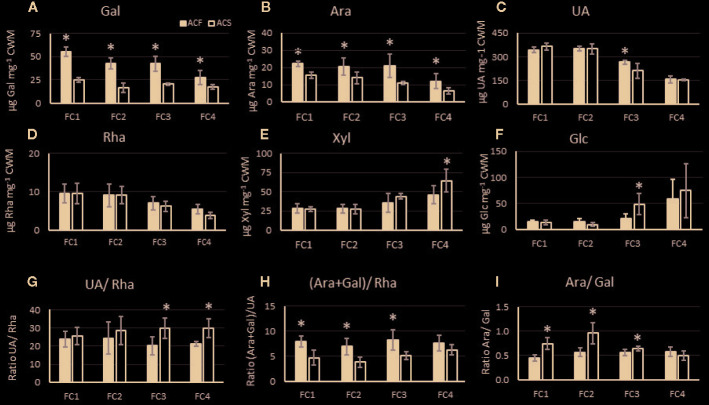
Composition of the main noncellulosic sugars in µg·mg^−1^ polysaccharide **(A–F)** and analysis of pectin side chains **(G–I)** of cell wall material (CWM) prepared from *Actinidia chinensis* var. *chinensis* fast softening ‘AC-F’ (black bars) and slow softening ‘AC-S’ (white bars) outer pericarp tissue over softening. Pectin side chain analysis is based on molar ratios of UA, rhamnose (rha), galactose (gal), and arabinose (ara). Degree of branching (UA : Rha) **(G)**, relative amounts of galactan and arabinan attached to the RG-I backbone (ara+gal/rha) **(H)**, proportions of arabinan *versus* galactan as RG-I side chains (ara/gal) **(I)** are based on mol%. Data are from CWM from outer pericarp of ‘season 2’, analyzed with three biological replicates using two technical replicates for neutral sugar and three technical replicates for UA analysis. Statistical significance of means between the two genotypes at the same firmness category (FC) is indicated by (*) (p <0.05). FC, firmness category.

In cell wall material derived from the outer pericarp of ‘season 2’ fruit, uronic acid (UA), galactose, xylose, and glucose were the cell wall main components in both genotypes (**Figure 5**). In ‘AC-F’, the galactose content decreased markedly over softening, indicating galactose loss, a key event in fruit softening. In ‘AC-S’, the galactose content was lower throughout softening and decreased at a much slower rate compared with ‘AC-F’ (**Figure 5A**). The fast softening ‘AC-F’ also had a significantly higher cell wall arabinose content compared with ‘AC-S’. Arabinose decreased in both genotypes over softening (**Figure 5B**). UA content was similar in both genotypes and decreased during softening, indicating pectin loss (**Figure 5C**). Rhamnose content was low and decreased over softening in both genotypes (**Figure 5D**). No significant difference was observed between genotypes at any softening stage. Xylose and glucose contents were similar between genotypes and increased during softening (**Figures 5E, F**). Mannose and fucose contents are not presented as they were present in very low amounts in both ‘AC-F’ and ‘AC-S’ (between 5 and 7 µg mannose and less than 1.9 µg fucose per mg CWM for both genotypes) with no significant differences between genotypes. **Supplementary Table S5** shows the sugar content of cell wall material from ‘season 1’, overall confirming observed trends in ‘season 2’ (**Figure 5**). The sugar composition of the cell wall extracts for both ‘season 1’ and ‘season 2’ is shown in **Supplementary Table S6**, indicating that there are differences in composition between ‘AC-F’ and ‘AC-S’ at different stages throughout softening.

Rhamnose, arabinose, galactose, and UA are components of RG-I pectin. Based on their molar ratio, pectin side chain analysis was carried out and revealed that length and branching of RG-I side chains were different between cell walls from ‘AC-F’ and ‘AC-S’ (**Figure 5**). The slow softening genotype ‘AC-S’ had higher UA/Rha and lower (Ara + Gal)/Rha ratios (**Figures 5G, H**), indicating the presence of smaller galactan and arabinan side chains in between longer HG stretches compared with ‘AC-F’. *Vice versa*, ‘AC-F’ had lower UA/Rha and higher (Ara + Gal)/Rha ratios, indicating larger galactan and arabinan side chains in between shorter HG stretches. Ara/Gal ratios were below 1 in both genotypes, indicating that galactose is prevalent in RG-I side chains. The Ara/Gal ratios were however higher in ‘AC-S’ compared with ‘AC-F’, indicating proportionally lower galactose content in the slow softening genotype (**Figure 5I**).

### Size Exclusion Chromatography Revealed That the Molecular Size Distribution of Pectic Polyuronides and Xyloglucan Is Different Between Both Genotypes During Softening

**Figure 6:**
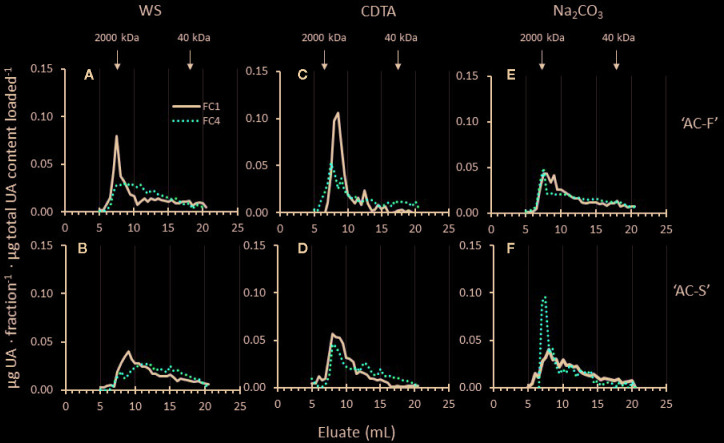
Polyuronide elution profiles after size exclusion chromatography of the water soluble (WS), CDTA- and Na_2_CO_3_-soluble cell wall extracts of *Actinidia chinensis* var. *chinensis* ‘AC-F’ **(A, C, E)** and ‘AC-S’ **(B, D, F)** at the unripe, firm stage FC1 (black lines) and at the soft stage FC4 (red dotted lines). Higher molecular weight peaks are to the left, while lower molecular weight peaks are to the right. FC, firmness category. 2,000 kDa and 40 kDa refer to elution of the molecular weight standards used. Samples (extracted from CWM of ‘season 1’ and ‘season 2’) were eluted on a Superose 6, 10/300 GL column.

During gel filtration chromatography, polyuronide elution profiles of the water-soluble and CDTA-soluble extracts of ‘AC-F’ (**Figures 6A, C**) showed that at FC1, ‘AC-F’ had a higher and sharper main peak between approximately 5 to 10 ml eluate compared with ‘AC-S’ (**Figures 6B, D**). In soft fruit at FC4 these peaks were reduced in height, indicating that pectin degradation was more pronounced in the fast softening genotype. Polyuronide degradation in the Na_2_CO_3_-soluble extract of both genotypes was negligible, but in the slow softening ‘AC-S’ genotype, a high molecular weight (MW) polyuronide peak appeared at FC4 when fruit was soft that was not present in ‘AC-F’ (**Figures 6E, F**).

**Figure 7:**
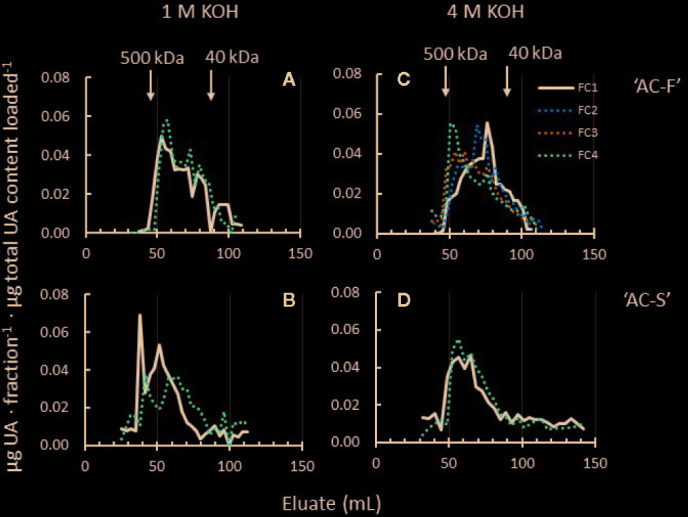
Polyuronide elution profiles after size exclusion chromatography of pectin in the 1 M KOH and 4 M KOH extracts of *Actinidia chinensis* var. *chinensis* fast softening genotype ‘AC-F’ **(A, C)** and slow softening ‘AC-S’ **(B, D)** at the unripe, firm stage FC1 (black lines) and at the soft stage FC4 (red dotted lines). Higher molecular weight peaks are to the left, while lower molecular weight peaks are to the right. Note that data from two additional firmness stages (FC2 and FC3) are shown in the 4 M KOH profile of ‘AC-F’. FC, firmness category. 500 kDa and 40 kDa refer to the elution of the molecular weight standards used. Samples (extracted from CWM of ‘season 1’ and ‘season 2’) were eluted on a Sephacryl S-300 column.

The 1 M and 4 M KOH extracts contain a mixture of pectin and hemicelluloses (**Supplementary Table S6**). Pectin polyuronide elution profiles indicated MW shifts in both extracts (**Figure 7**). Whereas the 1 M KOH-soluble pectin profiles of ‘AC-F’ showed little MW change, polyuronides in ‘AC-S’ showed a small but noticeable shift towards lower MW during softening, indicating pectin degradation. ‘AC-S’ polyuronides also start to elute earlier (at approximately 50 ml elution volume in ‘AC-F’ compared to ‘AC-S’ at approximately 35 ml), indicating the presence of a high MW polyuronide population in ‘AC-S’ that was not found in ‘AC-F’ (**Figures 7A, B**).

Whereas the MW distribution of the 4 M KOH soluble polyuronides in ‘AC-S’ did not change during softening, ‘AC-F’ elution profiles over the whole softening range indicated that polyuronides extractable in 4 M KOH increased in molecular size. At FC1, the main peak in ‘AC-F’ was eluted at around 80 ml eluate, indicating a lower MW than in ‘AC-S’ at FC1. Upon softening, the main peak gradually shifted towards larger polyuronide populations at FC2 and FC3. At FC4, the main polyuronide peak eluted at approximately 50 ml, a little larger than the corresponding peak of ‘AC-S’ at the same firmness (**Figures 7C, D**).

In kiwifruit, xyloglucan is the main neutral polysaccharide in the 1 M and 4 M KOH extracts, with only small amounts of mannan polysaccharides present that do not change MW during softening (Schröder et al., 2001). Hence, elution profiles of total carbohydrates monitored with the phenol-sulfuric assay represent mostly xyloglucan (**Supplementary Figure S1**). In ‘AC-F’, change in MW during softening was minimal in both the 1 M KOH and 4 M KOH extracts (**Supplementary Figures S1A, C**). Some xyloglucan MW reduction was seen in the 1 M KOH and 4 M KOH extracts of ‘AC-S’. Here, sharp peaks eluted between 50 and 60 ml in FC1 were reduced in height when fruits were ripe at FC4 (**Supplementary Figures S1B, D**).

### Gene Expression During Softening of ‘AC-F’ and ‘AC-S’

**Figure 8:**
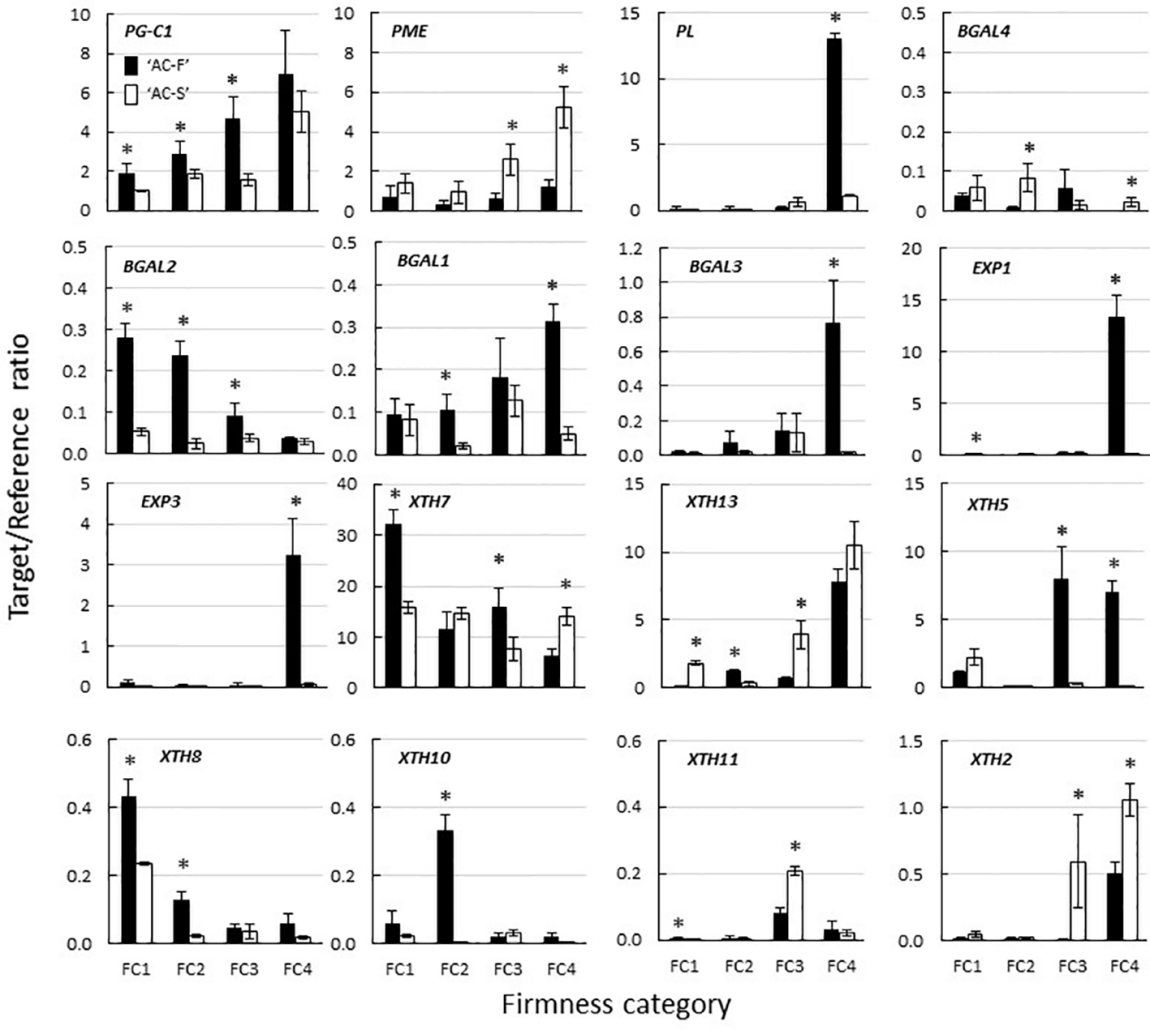
Expression of polygalacturonase (PG), pectin methylesterase (PME), pectate lyase (PL) *β*-galactosidase (BGal), expansin (EXP) and xyloglucan transglycosylase/hydrolase (XTH) genes during fruit softening of *Actinidia chinensis* var. *chinensis* fast softening genotype ‘AC-F’ and slow softening ‘AC-S’, determined using quantitative PCR. Values are means of three biological replicates using outer pericarp tissue from ‘season 2’ with technical replicates carried out in quadruplicate ± standard error. A two-tailed *t*-test was used to calculate the significance of sample means, and differences were deemed significant if *P* < 0.05. (*) denotes a significant difference between ‘AC-F’ and ‘AC-S’ at the same firmness category (*P* < 0.05). FC, firmness category. Note the different scales.

Although three PG cDNA clones (*PG-A, PG-B, and PG-C*) have been characterized in ripening kiwifruit, *PG-C1* (*Acc13940.1*) is the predominant gene expressed in softening fruit (Wang et al., 2000). Its expression increased throughout softening in both genotypes; however, expression was significantly higher in ‘AC-F’ than in ‘AC-S’ at FC1, FC2, and FC3. The *PME1* gene investigated in this study (*Acc29729.1*) was chosen based on its high expression in ripening fruit (Fullerton, 2015). In ‘AC-F’, expression of *PME1* did not change significantly over softening, whereas in ‘AC-S’ it increased significantly (**Figure 8**). The ripening-related *PL1* gene *(Acc18073.1)* (Atkinson et al., 2011) was also highly expressed in ‘AC-F’ at FC4 but not in ‘AC-S’ (**Figure 8**).

Four BGal genes (*BGAL1–4; BGAL1—Acc12764.1, BGAL2—Acc01038.1, BGAL3—Acc05054.1, BGAL4—Acc25938.1*) were also selected based on Fullerton (2015). With the exception of *BGAL4*, the fast softening ‘AC-F’ generally had a higher expression of BGal genes than ‘AC-S’. Expression of *BGAL4* varied over softening in ‘AC-F’ but was significantly higher in ‘AC-S’ at FC2 and FC4 and lower at FC3 compared with ‘AC-F’. Expression of *BGAL2* in ‘AC-F’ decreased during softening, whereas expression did not change significantly in ‘AC-S’. Expression of *BGAL2* was significantly higher in ‘AC-F’ at FC1, FC2, and FC3 compared with ‘AC-S’. The expression of *BGAL1* and *BGAL3* increased in ‘AC-F’ over softening while remaining relatively steady in ‘AC-S’ (**Figure 8**).

Forty-seven *EXP* gene models have been identified and systematically numbered in the manually annotated kiwifruit genome (Pilkington et al., 2018). Eight of these EXP genes had been identified as being expressed in *Actinidia* fruit (Fullerton, 2015). The expression of *EXP1 (Acc08682.1)* and *EXP3 (Acc27868.1)* was low in ‘AC-S’ during softening, while expression increased significantly in ‘AC-F’ at FC4 (**Figure 8**). Expression of *EXP2 (Acc31871.1), EXP4 (Acc14128.1), EXP5 (Acc33002.1), EXP6 (Acc30734.1), EXP7 (Acc32338.1)*, and *EXP8 (Acc01142.1)* was very low and showed no significant difference between genotypes.

Thirty-five *XTH* gene models and two partial models were identified (**Supplementary Figure S2**) and systematically numbered (**Supplementary Table S7**) in the manually annotated kiwifruit genome, including 14 *XTHs* previously shown to be expressed in the fruit (Atkinson et al., 2009). In ‘AC-F’ and ‘AC-S’, *XTH7* (*Acc00022.1*) was the most highly expressed gene. At FC1, *XTH7* expression was significantly higher in ‘AC-F’. At FC2, *XTH7* expression was not significantly different between the genotypes, while ‘AC-F’ had significantly higher expression at FC3, and ‘AC-S’ had significantly higher expression at FC4. The expression of *XTH13* (*Acc28326.1*) increased in both genotypes over softening and was higher in the slow softening ‘AC-S’ at all softening stages except FC2. *XTH5* (*Acc14348.1*), *XTH8* (*Acc08788.1*), and *XTH10* (*Acc00888.1*) were expressed at medium to low levels in the fast softening ‘AC-F’ but in lower levels in ‘AC-S’. In ‘AC-F’, *XTH5* was primarily expressed later in softening at FC3 and FC4, whereas *XTH8* and *XTH10* were expressed mostly in the early stages of softening at FC1 and FC2. Expression of *XTH11* (Acc18577.1) and *XTH2* (A’cc00504.1) was low in both genotypes. These genes were predominantly expressed in ‘AC-S’ later in softening at FC3 and FC4 (**Figure 8**).

### There Are Marked Differences in XTH Enzyme Activities Between Genotypes ‘AC-F’ and ‘AC-S’

**Figure 9:**
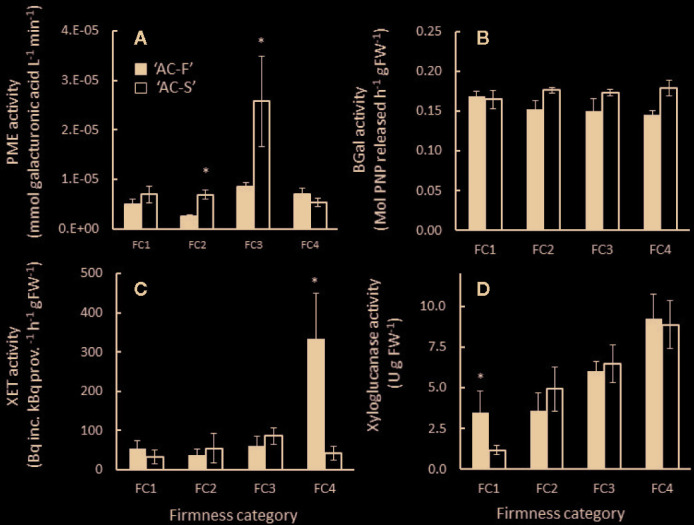
**(A)** Pectin methylesterase (PME), **(B)** *β*-galactosidase (BGal), **(C)** xyloglucan endotransglycosylase (XET) and **(D)** xyloglucanase activity in *Actinidia chinensis* var. *chinensis* fast softening genotype ‘AC-F’ (black bars) and slow softening ‘AC-S’ (white bars) during softening. Values are means of three biological replicates from ‘season 2’, each carried out in triplicate or quadruplicate ± S.D. A two-tailed *t*-test was applied to the enzyme activity results, and (*) denotes a significant difference between ‘AC-F’ and ‘AC-S’ at the same firmness category (*P* < 0.05). FC, firmness category.

The majority of PME activity was detected in the HS fractions, indicating that the enzyme is cell wall-bound. In ‘AC-F’, activity was low throughout softening. PME activity in ‘AC-S’ was significantly higher during rapid softening at FC2 and FC3 but similar at FC1 and FC4 compared with ‘AC-F’ (**Figure 9A**).

The majority of BGal activity was detected in the LS extracts, indicating that BGal was not cell wall-bound. BGal activity was similar in both genotypes without significant differences. In ‘AC-F’, BGal activity decreased slightly, while in ‘AC-S’ it did not change significantly over softening (**Figure 9B**). The immunoblots after hot SDS extraction of fruit tissue revealed several immuno-positive BGal bands in both genotypes, indicating that BGal in kiwifruit is present in multiple isoforms. A band detected at 59 kDa in both genotypes corresponds to the size of the BGal previously reported for kiwifruit (Ross et al., 1993). Both genotypes also showed a strong immune-positive band at about 67 kDa with increasing intensity over softening. In ‘AC-F’, two more bands were detected at approximately 46 and 50 kDa (**Supplementary Figures S3A, B**).

XET activity was similarly low in both LS and HS fractions in both genotypes during softening; however a significant increase in XET activity was observed in soft ‘AC-F’ fruit at FC4. The activity was extracted in the HS fraction, indicating it was cell wall-bound (**Figure 9C**). Xyloglucanase activity was predominantly detected in the LS extract, showing that the enzyme activity was soluble and not cell wall-bound. In both genotypes, xyloglucanase activity increased from FC2 to FC4 with no significant difference between ‘AC-F’ and ‘AC-S’ at similar firmness category. At FC1, however, xyloglucanase activity in the fast softening ‘AC-F’ was significantly higher than that of ‘AC-S’ (**Figure 9D**). After hot SDS extraction of fruit tissue, immunoblots using antibodies generated against XTH7 protein from kiwifruit revealed an immuno-positive XTH band at around 30 kDa in ‘AC-F’ that decreased in intensity over softening, whereas in ‘AC-S’, only a weak band was detectable at FC1 and a faint band at FC2 (**Supplementary Figures S3C, D**).

Expansin and polygalacturonase activity were not measured, but immunoblots raised against the gene products of *EXP3* or *PG-C1* from kiwifruit showed immune-positive bands. Expansin EXP3 from kiwifruit showed a broad immune-positive band at about 24 kDa in both genotypes. The intensity of this band remained constant from FC1 to FC3. At FC4, the band increased in intensity in ‘AC-F’ but decreased in ‘AC-S’. In ‘AC-F’ at FC4, other immune-positive bands around 23 kDa and 18 kDa appeared (**Supplementary Figures S3E, F**). Immunoblots using antibodies raised against kiwifruit PG-C protein were detected in both genotypes when fruits were ripe (**Supplementary Figure S3G**), confirming the observations of Bonghi et al. (1996) who detected PG activity only in ripe kiwifruit. The MW of kiwifruit PG is not known for certain, but the immune-positive band at *ca.* 37 kDa coincides with the predicted size of the full-length gene after processing

### Recombinant Proteins XTH5, XTH7, and XTH13 Have Differential Transglycosylation and Hydrolysis Activities

As there was differential XET and xyloglucanase activity between the genotypes during softening, XTH genes *XTH5*, *XTH7*, and *XTH13* were expressed in *E. coli*, and the recombinant proteins were tested for their ability to act as transglycosylases of xyloglucan and their ability to hydrolyze xyloglucan, therefore acting as xyloglucanase. These genes were chosen because they were highly expressed in both genotypes during softening.

**Figure 10:**
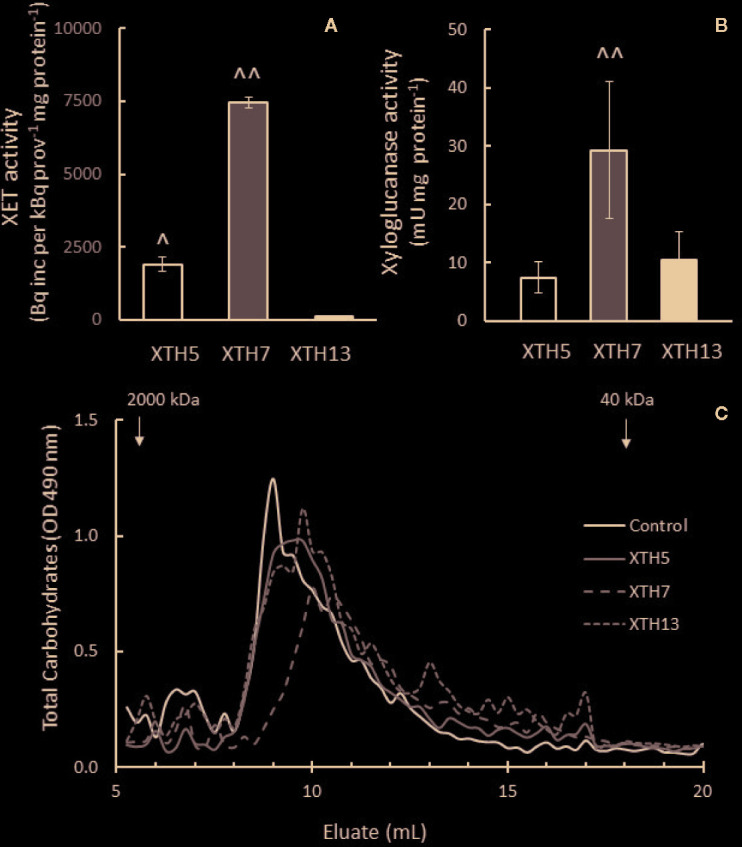
Enzyme activity of recombinant kiwifruit xyloglucan transglycosylase/hydrolase (XTH) XTH5, XTH7, and XTH13 proteins. **(A)** Xyloglucan endotransglycosylase (XET) activity **(B)** Xyloglucanase activity of the recombinant proteins. A two-tailed *t*-test was used to calculate the significance of sample means, and differences were deemed significant if *P* < 0.05. (^), indicates a significantly higher XET or xyloglucanase activity of XTH5 compared to XTH13, and (^^) a significantly higher XET or xyloglucanase activity of XTH7 compared to XTH5 or XTH13. **(C)** Total carbohydrate elution profile after size exclusion chromatography of tamarind xyloglucan alone (control) or incubated with XTH recombinant proteins on Superose 6, 10/300 GL. Higher molecular weight peaks are to the left, while lower molecular weight peaks are to the right. Recombinant XTH proteins were extracted from two different *E. coli* cultures and assayed three times per extract for XET and twice per extract for xyloglucanase activity.

In XET assays, XTH5 and XTH7 acted as transglycosylases, confirming the results for these enzymes reported by Atkinson et al. (2009). XTH13, however, did not exhibit transglycosylase activity even after prolonged assay times of 48 h. On a per mg protein basis, the recombinant XTH7 showed about four times higher transglycosylase activity than XTH5 (**Figure 10A**). All three recombinant proteins were able to hydrolyze xyloglucan, with XTH7 being significantly more active than either XTH5 or XTH13 in the gel diffusion assay, based on equivalent protein loadings (**Figure 10B**). The hydrolytic action was confirmed by size exclusion chromatography, where the xyloglucan elution profile after incubation with XTH7 showed the greatest shift towards smaller molecular size products compared with the xyloglucan control that was incubated with buffer only (**Figure 10C**). Elution profile shifts of xyloglucan after incubation with XTH5 and XTH13 are noticeable mainly in the reduction of the height of the main peak between 8 and 9 ml eluate and a shift towards smaller MW to the right, compared with the control.

## Discussion

In this study, two related genotypes of *Actinidia chinensis* var. *chinensis* were investigated; genotype ‘AC-F’ which softened rapidly within six days at ambient temperature and genotype ‘AC-S’, which took 13 days to soften to the same firmness. Starting from FC1, the longer softening time of ‘AC-S’ seems to be due to a slower transition between all firmness categories, including the rapid softening phase (FC2 and FC3) and the edible phase when fruit is ripe (FC4). Although both genotypes produced ethylene once the fruits were eating-soft at FC4, the slow-softening ‘AC-S’ produced on average more ethylene than the fast-softening ‘AC-F’. This indicates that endogenous ethylene production at this firmness stage is not correlated with the overall softening rate. In apple, Ng et al. (2013) found that the internal ethylene concentrations were similar between two apple cultivars even though the rates of softening were different, suggesting it is more than just ethylene regulating the rate of softening. The progression of ripening in both genotypes was consistent with other aspects of ripening, such as the increase in SSC and decrease in starch (as observed by a decrease in the yield of the DMSO soluble fractions) as described in Beever and Hopkirk (1990). While early studies suggested that starch may be involved in determining softening rate (MacRae et al., 1992), more recent data around starch and DM suggest that these alone do not have a large impact on softening rate (Woodward, 2007). Additionally, Burdon et al. (2017) showed variations in softening rates among selections and cultivars that had a similar DM content.

The outer pericarp tissues of both ‘AC-F’ and ‘AC-S’ contain large and small parenchyma cells, an arrangement which is typical for kiwifruit (Hallett et al., 1992). Staining with toluidine blue decreased in both cell types during softening. ‘AC-S’ retained more stainability at FC4, with the small cells still showing some staining, especially around plasmodesmatal pit fields, whereas in ‘AC-F’ at the same firmness, tissue connectivity seems to have been lost. This suggests that although fruits of both genotypes had the same firmness, the cell walls in ‘AC-S’ had greater cell wall and tissue integrity compared with ‘AC-F’. The small cells in ‘AC-F’ appeared to be only about half the size of those of ‘AC-S’. For apple it has been reported that cultivars with larger cells have better keeping qualities and ripen more slowly, possibly due to a lower respiratory rate compared to higher respiration in smaller cells (Smith, 1940; Nelmes and Preston, 1968).

Sugar compositional analyses and comparison of yields of CWM indicated that both genotypes show typical cell wall changes during kiwifruit softening such as loss of UA, galactose and arabinose over softening (Schröder and Atkinson, 2006), however at different rates or at different time points. Sugar compositional analyses of both genotypes also showed that the sugar composition of noncellulosic monosaccharide residues in the cell wall material (**Figure 5**) of ‘AC-F’ and ‘AC-S’ was different throughout softening when compared at the same firmness stage. This means that although fruits were compared at the same firmness, the cell walls of the two genotypes were different at each firmness stage, suggesting that the structure of the cell wall of each genotype may be a key determinant of the softening rate.

During pectin solubilization, pectin that is bound to the cell wall becomes freely soluble and extractable with water. However, unlike in apple where less pectin solubilization was associated with a slower softening rate (Ng et al., 2015), the yield of pectin in the water-soluble extract was less in the fast softening ‘AC-F’, compared with ‘AC-S’ (**Supplementary Table S3**). As the cell wall extracts of ‘AC-F’ and ‘AC-S’ were compared at the same firmness, this implies that for ‘AC-S’ to achieve the same firmness as ‘AC-F’, more pectin had to be solubilized from the cell wall.

The MW range of water-soluble pectin is likely to be an important factor influencing the softening rate as it can be assumed that the greater the loss of large MW pectin from the cell wall into the ‘freely soluble wall space’, the greater impact the loss would have on the structural integrity of the cell wall. Gel filtration chromatography showed that high MW populations were present in the water-soluble extract of firm ‘AC-F’ fruit but not in ‘AC-S’. Moreover, side chain analysis indicated that pectin in the water-soluble extract from ‘AC-F’ had larger galactan and arabinan side chains compared with AC-S’. Once these longer pectin chains including larger side chains are solubilized and “removed” from the cell wall network, this could contribute to a weaker cell wall structure and a faster softening rate in ‘AC-F’, especially as cell wall porosity might be increased (Somerville et al., 2004), thereby giving cell wall enzymes faster access.

Although pectin solubilization is a central part of fruit softening of all fleshy fruit, the mechanism by which this occurs is largely unknown. In strawberry, Posé et al. (2013) suggested that the transition of Na_2_CO_3_-soluble pectin becoming water-soluble pectin was correlated with high PG activity. Pectin solubilization, PG expression and PG activity were also positively correlated in apple. In transgenic apples where expression of the *MdPG1* gene was downregulated, pectin solubilization was reduced (Atkinson et al., 2012), whereas overexpression of the *MdPG1* gene in transgenic apple leaves led to increased pectin solubilization (Atkinson et al., 2002). In ‘AC-F’ and ‘AC-S’ however, this correlation was not observed. In both genotypes, PG protein was only detectable when fruits were ripe at FC4 (**Supplementary Figure S3**), while pectin solubilization had already started at FC1. Furthermore, *PG-C1* expression was consistently higher in ‘AC-F’ compared with ‘AC-S’ throughout softening, yet the yields of water-soluble pectin were lower (**Supplementary Table S3**). As suggested for persimmon (Cutillas-Iturralde et al., 1993), there might be a PG-independent pectin solubilization mechanism present in kiwifruit. Some of this solubilization may be attributed to the activity of pectate lyase as the expression of a *PL* gene was detected in both genotypes, albeit in low levels early in softening. The role which pectate lyase plays in kiwifruit softening remains largely unknown. A study by Prakash et al. (2017) which used an antibody directed against kiwifruit pectate lyase did not reveal the presence of protein in the kiwifruit genotypes analyzed, despite gene expression being present. In this current study, while the expression of *PL* was significantly higher in ‘AC-F’ at FC4, it is unlikely this would influence pectin solubilization or the softening rate, as much of these processes had already occurred by this point.

Galactose and arabinose are present in cell walls mostly in the form of long and branched arabinan and galactan side chains of RG-I. In ‘Hayward’ kiwifruit, galactose loss from galactan side chains is a major event in kiwifruit softening, while arabinans and arabinose loss seem to play a minor role (Schröder and Atkinson, 2006). In this study, galactose loss in cell walls was observed in both genotypes; however the galactose content and the rate of galactose loss in ‘AC-S’ were much lower than that of ‘AC-F’. Galactans are able to anchor spectin to the wall through binding to either xyloglucan or cellulose, and loss of galactose from these side chains has been found to reduce cell wall strength and increase wall porosity (Renard et al., 1991; Popper and Fry, 2005; Zykwinska et al., 2005; Zykwinska et al., 2006; Zykwinska et al., 2007).

Immunolabeling with galactan-specific LM5 showed a similar distribution of the epitope in ‘AC-F’ and ‘AC-S’ at FC1 and FC2, but at FC4, labeling was more intense in the large cells of ‘AC-F’ whereas in ‘AC-S’ it was more intense in the small cells. The structural integrity of the tissue is likely to be based on the strength and connectivity of the small cells as they make up about 60% of the outer pericarp and provide the matrix in which the large cells are embedded (Hallett et al., 1992). In pea cotyledons (McCartney et al., 2000), potato (Ulvskov et al., 2005), tomato (Smith et al., 2002) and ‘Hayward’ kiwifruit (Redgwell et al., 1997), reduced galactose content was correlated with a decrease in wall strength and tissue firmness. In potato tubers, a reduction in galactans resulted in increased solubility of pectin and wall porosity (Sørensen et al., 2000). Hence the loss of galactose in the small cells of ‘AC-F’ could result in more porous walls, thus providing easier access for cell wall degrading enzymes to their substrates (McCartney et al., 2000). These results suggest that in kiwifruit, with its unusual large cell/small cell arrangement, it is just as important to know from which cell type the galactose is lost as it is to know from which cell wall extract or at which time point during softening it is lost.

During softening there was little increase in BGal activity in either genotype despite an increase in intensity of the immune-positive bands in both genotypes over softening and a lower number of bands (**Supplementary Figure S3**). This suggests that the measured BGal activity in both genotypes might not be completely cell wall-related. In tomato, while total BGal activity was high and did not change significantly during ripening, transcripts derived from seven BGal genes detected during fruit development showed different patterns of expression (Carey et al., 1995; Carrington and Pressey, 1996; Smith and Gross, 2000). Smith et al. (2002) found that the downregulation of *TBG4*, a tomato BGal gene expressed early in ripening and increasing over four-fold during ripening in wild-type (Carey et al., 1995) resulted in firmer fruit compared with the control. Similarly, Paniagua et al. (2016) found that downregulation of a strawberry BGal *FaβGal4* gene resulted in fruit with a lower BGal expression, a higher galactose content, lower polyuronide solubilization, and a higher fruit firmness compared to the control fruit at the ripe stage. A decrease in wall porosity caused by the downregulation BGal enzyme activity may have led to the obstruction of access of other cell wall degrading enzymes, hindering the depolymerization of structural polysaccharides (Brummell and Harpster, 2001). Lower cell wall-related BGal enzyme activity, decreased cell wall porosity, and more difficult access of cell wall enzymes to their substrates could be contributing to the slow softening rate of ‘AC-S’.

In terms of pectin degradation, no large shifts towards small MW populations were observed in elution profiles in either genotype. Instead, high-MW populations either diminished over softening (*e.g.* in the WS- and CDTA-extracts of ‘AC-F’ or in the 1 M KOH extract of ‘AC-S’), or appeared over softening, (*e.g.* in the Na_2_CO_3_-soluble pectin of ‘AC-S’ or the 4 M KOH-soluble pectin in ‘AC-F’). Reductions in peak height of high-MW populations without a shift towards smaller MW populations might be caused by the removal of galactan side chains through galactose loss, or the increase in MW could be caused by reallocation of pectin during softening. Studies on kiwifruit and tomato have shown evidence of such reallocation, resulting in clear MW shifts towards larger molecules (Redgwell et al., 1992; Brummell and Labavitch, 1997). Solubilization and reallocation of very tightly bound pectin could influence the structural integrity of the cell wall. In ‘AC-F’, the 4 M KOH soluble pectin had a lower MW distribution than ‘AC-S’ at FC1, suggesting that the cell wall structure may have been already loosened and weakened, allowing greater access to cell wall degrading enzymes. As ‘AC-F’ fruit progressively softened, larger MW pectin molecules from the cell wall residue may have become gradually less tightly bound, and released upon extraction with 4 M KOH. In ‘AC-S’, the MW distribution of 4 M KOH extracted pectin did not change during softening, indicating that pectin molecules remained tightly bound. Cell walls were likely stronger and less ‘loosened’, giving cell wall degrading enzymes less space to move and bind, resulting in a slower softening rate. Increasing evidence of covalent links between pectin and cellulose (Park and Cosgrove, 2012; Broxterman and Schols, 2018) could indicate a difference in the way pectin, xyloglucan and cellulose are interacting within the cell walls in each genotype, with a stronger association between pectin and cellulose in ‘AC-S’ compared to ‘AC-F’. This could make this pectin harder to release and break down, thereby contributing to its slower softening rate.

There were differences in the xyloglucan-domain of the cell wall between both genotypes. At FC4, immunolabeling showed that the XXXG epitope of xyloglucan was mainly located in the large cells in ‘AC-F’, while in ‘AC-S’ both the large and small cells showed labeling. As xyloglucan contributes to the structural integrity of the cell wall by holding cellulose microfibrils in place in the cell wall matrix (Cosgrove, 2005), it can be assumed that due to higher xyloglucan content in the cell wall of both small and large cells, ‘AC-S’ is likely to show greater resistance to changes to the xyloglucan–cellulose structure during softening that are mediated through the action of XET and xyloglucanase activity.

XTH proteins have different donor and acceptor substrate specificities and can act as dual activity enzymes, having either predominantly XET or xyloglucanase activity (Strohmeier et al., 2004). In ‘AC-F’ and ‘AC-S’, multiple isoforms of XTH were present as shown by immunoblotting and gene expression analysis. Significant XET activity was only detected in ripe ‘AC-F’ fruit while both genotypes exhibited xyloglucanase activity. Xyloglucanase activity was detected later in softening in ‘AC-S’ fruit than in ‘AC-F’ where xyloglucanase activity was already present in firm unripe fruit.

To relate XET and xyloglucanase activity with gene expression, kiwifruit genes *XTH5*, *XTH7*, and *XTH13* were expressed in *E. coli*. The expression of *XTH5* (previously *AdXET5,* as reported by Schröder et al., 1998) increased in the outer pericarp tissue of ‘AC-F’ during softening but remained low in ‘AC-S’. *XTH13* showed overall higher expression in ‘AC-S’, and *XTH7* was the main gene expressed in both genotypes. Whereas recombinant proteins XTH7 and XTH5 showed both XET and xyloglucanase activity, and hence are true dual activity enzymes, XTH13 showed xyloglucanase activity only. Interestingly, despite the presence of at least three XTH enzymes with xyloglucanase activity *in vitro*, there was little change in MW of xyloglucan in either 1 M or 4 M KOH extracts in both genotypes. Redgwell and Fry (1993) reported an increase in XET activity in ethylene-treated ‘Hayward’ during softening, which was positively correlated with a reduction in the relative MW of xyloglucan (Schröder and Atkinson, 2006). However, overexpression of an XET gene from tobacco in tomato resulted in an increase in average MW of xyloglucan and firmer tomatoes than the wild type (Miedes et al., 2010) while downregulation of a tobacco XTH gene in tobacco also led to an increase in MW of xyloglucan in leaf midribs (Herbers et al., 2001). It is possible that in kiwifruit, the xyloglucan substrates for XET or xyloglucanase activity may not have been extracted with 1 M or 4 M KOH but remained in the insoluble, cellulose-rich cell wall residue left after sequential extraction. Poplars overexpressing a xyloglucanase provide evidence that this enzyme is able to act on xyloglucan that is very tightly bound to cellulose, as the crystalline region of cellulose microfibrils in the xylem of the transgenic trees was highly degraded, compared to wild type (Kaida et al., 2009). Reduction of the MW of very tightly bound xyloglucans could weaken the cell wall and contribute to a faster softening rate of ‘AC-F’.

Expansins may play a role in the loosening of the cellulose–xyloglucan hydrogen bonds, thereby increasing the accessibility of XTH to its xyloglucan substrate *in vivo* (Cosgrove et al., 2002). When bound to cellulose, xyloglucan is inaccessible to xyloglucanase (Pauly et al., 1999), but the presence of expansin allows the loosening of the hydrogen bonds or sufficient separation between the chains to allow the enzyme to bind. Brummell et al. (1999) showed that overexpression of LeEXP1 in green tomato fruit caused depolymerization of xyloglucans, presumably mediated by xyloglucanase or other hydrolases such as endoglucanase. ‘AC-F’ had a higher number of immune-positive bands reacting with the expansin antibody, and presumably a higher expansin activity compared to ‘AC-S’. This could have increased the binding capacity and *in vivo* activity of XTH by enabling access to its xyloglucan substrate early in softening, thereby contributing to its faster softening rate.

## Conclusions

The results from this study highlight the complexity of the fruit softening process, involving many factors such as differences in tissue structure, cell wall biochemistry, gene expression and enzyme activity between the fast and slow softening genotypes of *A. chinensis* var. *chinensis* (‘AC-F’ and ‘AC-S’). Analysis of the cell wall composition and sequential extracts, as well as changes in molecular weight during softening showed differences between genotypes at the same firmness. These data indicated that the different softening rates of ‘AC-F’ and ‘AC-S’ are not just a matter of increased or decreased gene expression and enzyme activity, but that there are differences in the cell wall structure even in the early stages of the softening process. These differences mostly seem to relate to wall strength and accessibility for enzymes, possibly due to differences in pore size of cell walls. From the firm, unripe stage onwards, ‘AC-F’ cell walls seemed more loosened, possibly giving cell wall degrading enzymes more space to move and act on their substrates, resulting in a faster softening rate. Cell walls of ‘AC-S’ on the other hand, seemed to be stronger and less loosened with possibly lower pore size and as a consequence, less easy to attack. Hence in ‘AC-S’, the time needed to soften and reach a certain firmness was much longer compared with ‘AC-F’.

## Data Availability Statement

The datasets generated for this study are available on request to the corresponding author.

## Author Contributions

CF, RS, IH, RA, and RJS conceived the project; RS, CF, RA, IH, and RJS wrote the manuscript; CF, RP, AN, and RS conducted the experiments.

## Funding

This work was funded by the NZ Ministry of Business, Innovation and Employment, and internal PFR funding derived in part from kiwifruit variety royalty income.

## Conflict of Interest

The authors declare that the research was conducted in the absence of any commercial or financial relationships that could be construed as a potential conflict of interest.

## Abbreviations

‘AC-F’, *Actinidia chinensis* fast softening genotype; ‘AC-S’, *A. chinensis* slow softening genotype; *α*-L-arabinofuranosidase, AFase; BGal, *β*-galactosidase; CDTA, trans-1,2-diaminocyclohexane-N,N,N′,N′-tetraacetic acid; CWM, cell wall material; DMSO, dimethyl sulfoxide; FC, firmness category; HG, homogalacturonan; PME, pectin methylesterase; PG, polygalacturonase; RG-I, rhamnogalacturonan-I; SSC, soluble solid concentration; UA, uronic acids; XG, xyloglucan; XTH, xyloglucan endotransglycosylase/hydrolase; XET, xyloglucan endotransglycosylase.
