# Supplementary material for: Fruit From Two Kiwifruit Genotypes With Contrasting Softening Rates Show Differences in the Xyloglucan and Pectin Domains of the Cell Wall
Source: Front Plant Sci. 2020 Jul 2;11:964. doi: 10.3389/fpls.2020.00964 (PMC7343912; doi:10.3389/fpls.2020.00964)

**Supplementary Figure S1** Elution profiles of total carbohydrates of 1 M KOH and 4 M KOH extracts after size exclusion chromatography of the fast softening *Actinidia chinensis* var. *chinensis* genotype 'AC-F' **(A, C)** and slow softening 'AC-S' **(B, D)** at the unripe, firm stage FC1 (black lines) and at the soft stage FC4 (dotted lines). Higher molecular weight peaks are to the left, while lower molecular weight peaks are to the right. The total carbohydrate (TC) profile was monitored using the phenol-sulphuric assay. FC, firmness category. 500 kDa and 40 kDa refer to the elution of the molecular weight standards used. Samples (extracted from CWM of ‘season 1’ and ‘season 2’) were eluted on a Sephacryl S-300 column.


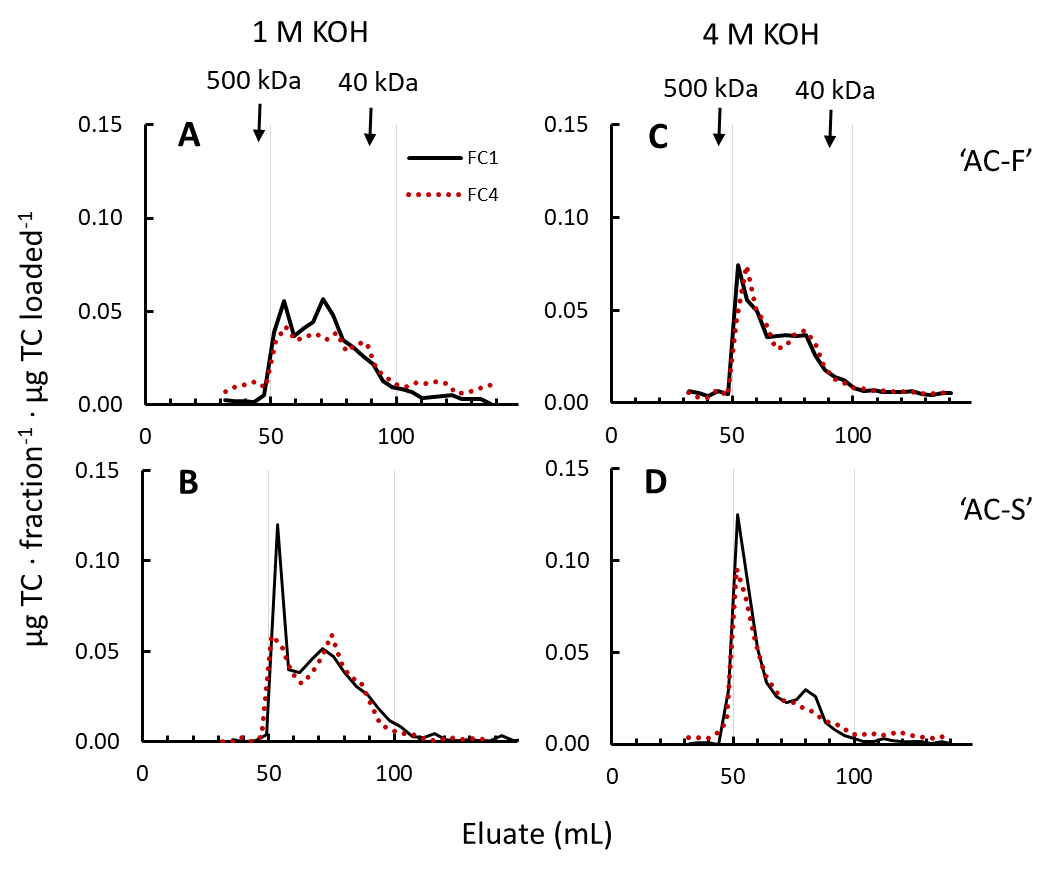

Supplement: Supplementary file 1 [file DataSheet_1.docx]
