# Supplementary material for: Fruit From Two Kiwifruit Genotypes With Contrasting Softening Rates Show Differences in the Xyloglucan and Pectin Domains of the Cell Wall
Source: Front Plant Sci. 2020 Jul 2;11:964. doi: 10.3389/fpls.2020.00964 (PMC7343912; doi:10.3389/fpls.2020.00964)

**Supplementary Figure S2** Phylogenetic analysis of Arabidopsis and kiwifruit XTH protein sequences. Reference Arabidopsis proteins are in blue and predicted XTH proteins from *Actinidia chinensis* var. *chinensis* gene models are in black. Amino acid sequences were aligned using the Geneious software package (https://www.geneious.com).The phylogenetic tree was produced using PHYML. Bootstraps values from 1000 iterations are given.


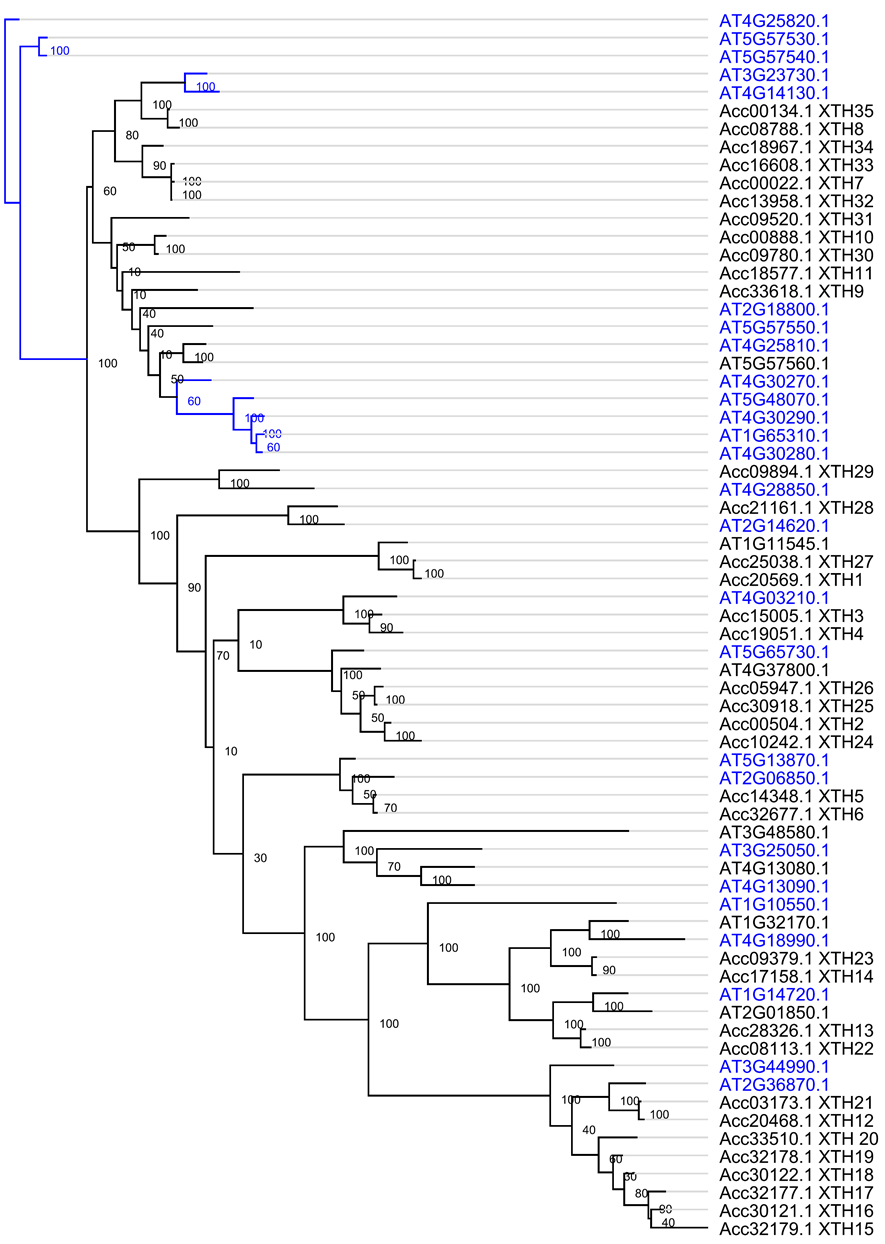

Supplement: Supplementary file 2 [file DataSheet_2.docx]
