# Supplementary material for: Fruit From Two Kiwifruit Genotypes With Contrasting Softening Rates Show Differences in the Xyloglucan and Pectin Domains of the Cell Wall
Source: Front Plant Sci. 2020 Jul 2;11:964. doi: 10.3389/fpls.2020.00964 (PMC7343912; doi:10.3389/fpls.2020.00964)
Supplement: Supplementary file 3 [file DataSheet_3.docx]

**Supplementary Figure S3** Western analyses of total protein extracts from *Actinidia chinensis* var. *chinensis* fast softening genotype ‘AC-F’ and slow softening genotype ‘AC-S’. Polyclonal antibodies used were β-galactosidase (BGal) from apple (A, B), xyloglucan endotransglycosylase/hydrolase (C, D) XTH7, expansin EXP3 (E, F) and polygalacturonase PG-C1 (G) from kiwifruit. Lane 1, Precision Plus Dual Protein Standard (BioRad); FC, firmness category. Arrows indicate the presence of immune-positive protein bands. Outer pericarp tissue was derived from ‘season 2’ fruit.

**
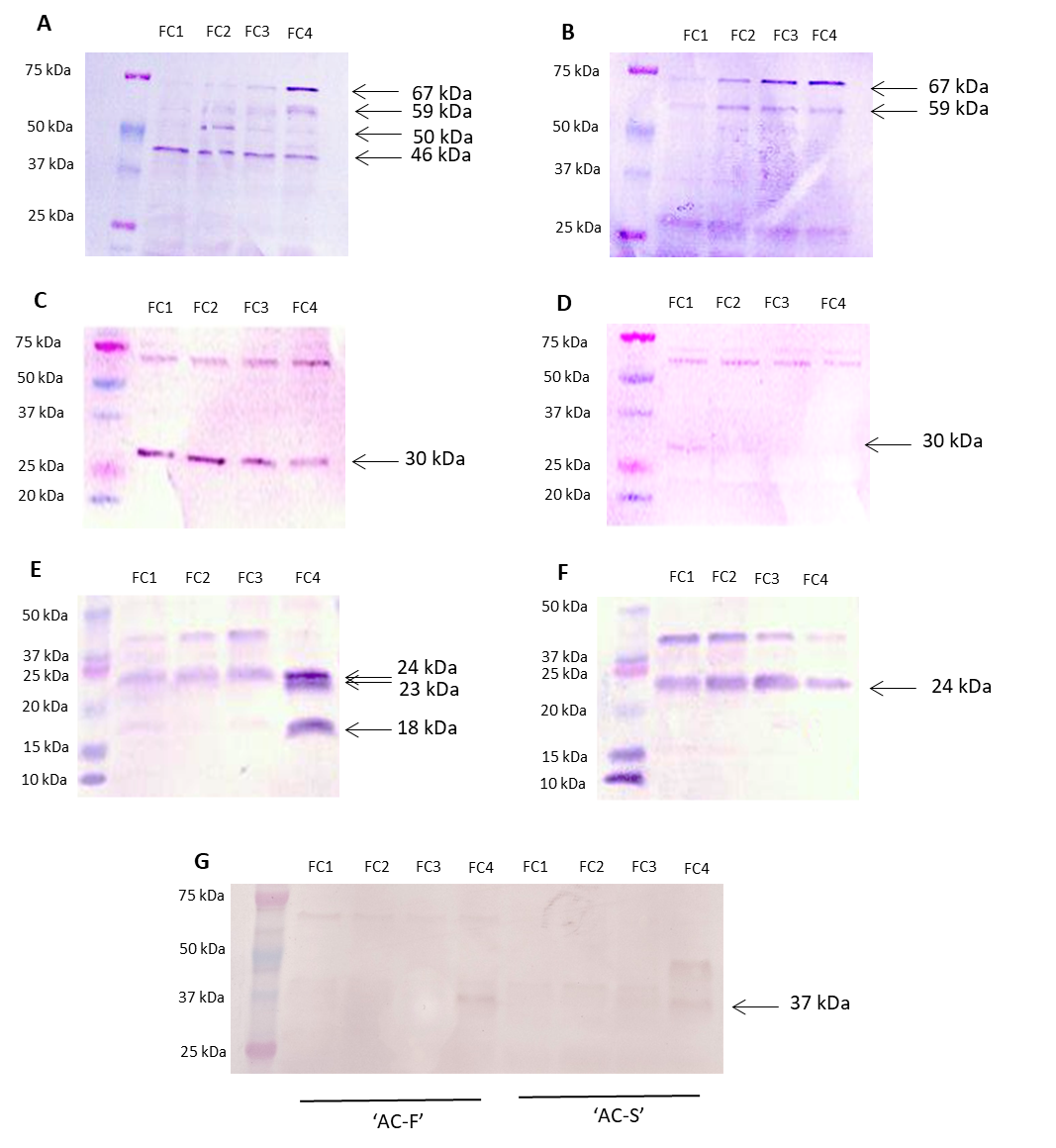
**

**Method:** Total protein was extracted by boiling 100 mg finely ground tissue in 0.5 mL of extraction buffer (0.1 M Bis Tris, 2 M glycerol, 0.3 M sodium dodecyl sulfate, 0.2 M dithiothreitol, 0.01% Brilliant Blue G) for 10 min. After centrifugation (10 min, 11,000xg), the supernatants (10 µL) were loaded onto 10% polyacrylamide gels. Protein gel electrophoreses, western blotting and staining were carried out as described in Prakash et al (2017) using polyclonal antibodies developed against BGal1 from apple (Atkinson et al. 2012), xyloglucan transglycosylase/ hydrolase XTH7, expansin EXP3 and polygalacturonase PG-C1 from kiwifruit (Prakash et al. 2017). Western blots were carried out twice, scanned with a high resolution scanner and images adjusted for brightness. Typical images of blots are shown.
