# Supplementary material for: Fruit From Two Kiwifruit Genotypes With Contrasting Softening Rates Show Differences in the Xyloglucan and Pectin Domains of the Cell Wall
Source: Front Plant Sci. 2020 Jul 2;11:964. doi: 10.3389/fpls.2020.00964 (PMC7343912; doi:10.3389/fpls.2020.00964)
Supplement: Supplementary file 4 [file Table_1.docx]

**Supplementary Table S1.** Primary monoclonal anti-rat antibodies, their targets, and dilution factors used for immunolabelling.

| **Antibody** | **Target** | **Dilution (v/v)** | **Reference** |
| --- | --- | --- | --- |
| JIM5 | Unesterified or partially esterified HG | 1:20 | Knox et al., 1990; Planta 181: 512-521 |
| JIM7 | Methylesterified HG | 1:20 |  |
| LM5 | (1-4)-β-D-Galactan | 1:100 | Jones et al., 1997; Plant Physiol. 113: 1405-1412 |
| LM15 | XXXG motif of Xyloglucan | 1:40 | Marcus et al., 2008; BMC Plant Biol. 8: 60 |
