# Supplementary material for: Fruit From Two Kiwifruit Genotypes With Contrasting Softening Rates Show Differences in the Xyloglucan and Pectin Domains of the Cell Wall
Source: Front Plant Sci. 2020 Jul 2;11:964. doi: 10.3389/fpls.2020.00964 (PMC7343912; doi:10.3389/fpls.2020.00964)
Supplement: Supplementary file 5 [file Table_2.docx]

**Supplementary Table S2** Oligonucleotide primers for qPCR. Primers for xyloglucan transglycosylase/hydrolase (XTH) and kiwifruit actin primers (McAtee et al., 2015), expansin (EXP), β-galactosidase (BGAL), polygalacturonase (PG), pectin methyl esterase (PME) and pectate lyase genes. Fwd, forward primer; Rev, reverse primer.

| **Name** | **Accession number** | | **Primer sequence (5'-3')** | **Name** | **Accession number** | | **Primer sequence (5'-3')** |
| --- | --- | --- | --- | --- | --- | --- | --- |
| *XTH1* | Acc20569.1 | Fwd | CTCGTACAAGGACTTCAGCGTGGA | *EXP1* | Acc08682.1 | Fwd | AGGGTCACGACAACGGATGGAGT |
|  | Acc20569.1 | Rev | TGGATACACAGGCAGGGTAAGGGT |  | Acc08682.1 | Rev | CGAGCTAGAGAAGGTTTGGCCGA |
| *XTH2* | Acc00504.1 | Fwd | GCGGCGGCTTTCCACACTTA | *EXP2* | Acc31871.1 | Fwd | TCAATCAAGGGGTCAAAGACAGGGT |
|  | Acc00504.1 | Rev | TGGGACTCCTCTGGCTTCATTGTT |  | Acc31871.1 | Rev | TTTGGCCCCAATTCCTCGACA |
| *XTH3* | Acc15005.1 | Fwd | GAGCGGGGAGCCTTATCTGGTT | *EXP3* | Acc27868.1 | Fwd | CATGTCAAGGAACTGGGGCCA |
|  | Acc15005.1 | Rev | ACCCACACCGTTCACATACACGTT |  | Acc27868.1 | Rev | GATGGTTCTTCCGTCACTGGTGGT |
| *XTH4* | Acc19051.1 | Fwd | GGGTCAGGTCCAAGCACATGGT | *EXP4* | Acc14128.1 | Fwd | TCTGCCCACCAAACAGTGAAGGA |
|  | Acc19051.1 | Rev | CACCGGAAACCTAGTCTTGTCGCT |  | Acc14128.1 | Rev | GCAATGTGTTGGAAGACAGGCTGA |
| *XTH5* | Acc14348.1 | Fwd | CGAGACCTCGACGCCTTCCA | *EXP5* | Acc33002.1 | Fwd | CCTCCAAACAATGCCTTACCAAACA |
|  | Acc14348.1 | Rev | CAGGAGGCATGGTGGGGTATCTT |  | Acc33002.1 | Rev | TGTTGGAAGACAGGCTGAGAGAGGT |
| *XTH6* | Acc32677.1 | Fwd | GCTCCAGTGGGTACGCTCCAAA | *EXP6* | Acc30734.1 | Fwd | GGGGCAAAATTGGCAAAGCA |
|  | Acc32677.1 | Rev | ACGTCCCGATCTCTGCTGCATT |  | Acc30734.1 | Rev | GGAGACCACACTGCGACCATCA |
| *XTH7* | Acc00022.1 | Fwd | CAAGGAGGGAGAGTGAAGACGGATT | *EXP7* | Acc32338.1 | Fwd | GGCGCAGGGAATATAGTGAGGGT |
|  | Acc00022.1 | Rev | AAGAGTGTACGCAAGCATTGGCATT |  | Acc32338.1 | Rev | GTTCCGGCTCATGCTCATCCA |
| *XTH8* | Acc08788.1 | Fwd | CATGTAGTCCCCGCTCTTGCACTT | *EXP8* | Acc01142.1 | Fwd | GGGGGCAAAACTGGCATATAAACA |
|  | Acc08788.1 | Rev | CCATCTCAGTCGCCTTCGGCTATA |  | Acc01142.1 | Rev | CGGTGAGTTCAAATGCAAGAGGCT |
| *XTH9* | Acc33618.1 | Fwd | AGGCTCCAGTGGGTGCAGAAGAA | *BGAL1* | Acc12764.1 | Fwd | CAGTGATGTGAATCTGAGACCTGG |
|  | Acc33618.1 | Rev | AACATTCTGGAGGGAGGCCCTGT |  | Acc12764.1 | Rev | CCCTTCTTCCCTCATTGAGACC |
| *XTH10* | Acc00888.1 | Fwd | GCTCTTCTTCCTCCTCGTGTTCCAA | *BGAL2* | Acc01038.1 | Fwd | GATGCCCCAGATCCAGTG |
|  | Acc00888.1 | Rev | GATCCTTTCTTGGCCGGTCGA |  | Acc01038.1 | Rev | GTAAACCAGCCACTCCAAG |
| *XTH11* | Acc18577.1 | Fwd | TCCTACAGAAAATTCAATGCTCGGG | *BGAL3* | Acc05054.1 | Fwd | GCAGGGACTCCTACGCAAAC |
|  | Acc18577.1 | Rev | GAGAGGAGGTTGAGGTTGAGGTTGG |  | Acc05054.1 | Rev | CTGTCAGAAACATGCCCACATAC |
| *XTH12* | Acc20468.1 | Fwd | AGCGGAGATGGGAGAATTATAGGGA | *BGAL4* | Acc25938.1 | Fwd | CCACCCATCTCCTCGAACAAG |
|  | Acc20468.1 | Rev | TGTGTTGGATCAAACCATAGGTGGA |  | Acc25938.1 | Rev | CCCTTACAAGTCATACAAATGCCTC |
| *XTH13* | Acc28326.1 | Fwd | CCCTCCGTCGGAATGTGCAA | *PG-C1* | Acc13940.1 | Fwd | AGGCAACAGCCCAAACACC |
|  | Acc28326.1 | Rev | GGCCCCCTCCGAATGTGACT |  | Acc13940.1 | Rev | CGACATTCTCGATCCACAAATTC |
| *XTH14* | Acc17158.1 | Fwd | AGTAGAGAGGCGGCAATTCAAGGAT | *PME1* | Acc29729.1 | Fwd | ACGACGTCGTTTGAAGGGGAAGCG |
|  | Acc17158.1 | Rev | CATGCGTGACACCTGGCCTTTT |  | Acc29729.1 | Rev | CCCCAACCGTCGTGAAGTTACCG |
| Actin | Acc05529.1 | Fwd | TGCATGAGCGATCAAGTTTCAAG | *PL* | Acc18073.1 | Fwd | TTCAATCACTTTGGGGAAGGC |
|  | Acc05529.1 | Rev | TGTCCCATGTCTGGTTGATGACT |  | Acc18073.1 | Rev | GGTCACCTCTTTGCTGAATCTGA |
| MDP0000173025 |  | Fwd | ′CCATATCCAGGCTTGCCTAA |  |  |  |  |
|  |  | Rev | ACGAGGGTAACCTCACATGC |  |  |  |  |
