# Supplementary material for: Fruit From Two Kiwifruit Genotypes With Contrasting Softening Rates Show Differences in the Xyloglucan and Pectin Domains of the Cell Wall
Source: Front Plant Sci. 2020 Jul 2;11:964. doi: 10.3389/fpls.2020.00964 (PMC7343912; doi:10.3389/fpls.2020.00964)
Supplement: Supplementary file 6 [file Table_3.docx]

**Supplementary Table S3.** Yields of cell wall material (CWM), water-soluble (WS) extract, and DMSO (dimethyl sulfoxide) extract. CWM, WS and DMSO values are means from 3 biological replicates of ‘season 2’ and are given on a mg∙g^-1^ FW basis. Statistical significance of yields between the two genotypes at the same firmness category (FC) is represented by grey shading (p <0.05).

|  |  | 'AC-F' | 'AC-S' |  |
| --- | --- | --- | --- | --- |
|  |  | (mg g^-1^ FW) | |  |
| CWM | FC1 | 9.1 | 10.6 |  |
|  | FC2 | 9.4 | 9.7 |  |
|  | FC3 | 8.1 | 6.5 |  |
|  | FC4 | 5.3 | 3.0 |  |
|  |  |  |  |  |
| WS | FC1 | 2.0 | 2.6 |  |
|  | FC2 | 2.4 | 3.1 |  |
|  | FC3 | 3.2 | 5.8 |  |
|  | FC4 | 6.4 | 8.1 |  |
|  |  |  |  |  |
| DMSO | FC1 | 37.6 | 69.1 |  |
|  | FC2 | 25.9 | 46.2 |  |
|  | FC3 | 25.8 | 16.1 |  |
|  | FC4 | 2.6 | 6.0 |  |
|  |  |  |  |  |
|  |  |  |  |  |
|  |  |  |  |  |
|  |  |  |  |  |
|  |  |  |  |  |
|  |  |  |  |  |
|  |  |  |  |  |
|  |  |  |  |  |
|  |  |  |  |  |
|  |  |  |  |  |
