# Supplementary material for: Fruit From Two Kiwifruit Genotypes With Contrasting Softening Rates Show Differences in the Xyloglucan and Pectin Domains of the Cell Wall
Source: Front Plant Sci. 2020 Jul 2;11:964. doi: 10.3389/fpls.2020.00964 (PMC7343912; doi:10.3389/fpls.2020.00964)
Supplement: Supplementary file 7 [file Table_4.docx]

**Supplementary Table S4.** Firmness (in N), soluble solids concentration (SSC; in °Brix) and endogenous ethylene production (in nmol ethylene∙kg fruit^-1^∙s^-1^) throughout softening during ‘season 1’. Statistical significance of values between the two genotypes at the same firmness category (FC) is represented by grey shading (p <0.05).

|  |  | ‘AC-F’ | ‘AC-S’ |
| --- | --- | --- | --- |
| Firmness | FC1 | 84 | 78 |
|  | FC2 | 55 | 52 |
|  | FC3 | 21 | 16 |
|  | FC4 | 4 | 5 |
| SSC | FC1 | 12.1 | 14.1 |
|  | FC2 | 15.1 | 16.8 |
|  | FC3 | 17.6 | 19.5 |
|  | FC4 | 19.6 | 19.1 |
| Ethylene | FC1 | 0 | 0 |
|  | FC2 | 0 | 0.01 |
|  | FC3 | 0.01 | 0.02 |
|  | FC4 | 1.37 | 0.34 |
| Dry Matter | FC1 | 20.7 | 19.9 |
