# Supplementary material for: Fruit From Two Kiwifruit Genotypes With Contrasting Softening Rates Show Differences in the Xyloglucan and Pectin Domains of the Cell Wall
Source: Front Plant Sci. 2020 Jul 2;11:964. doi: 10.3389/fpls.2020.00964 (PMC7343912; doi:10.3389/fpls.2020.00964)
Supplement: Supplementary file 8 [file Table_5.docx]

**Supplementary Table S5.** Non-cellulosic sugar composition of cell wall material (CWM) in µg∙mg^-1^ polysaccharide material in the anhydro (= polymeric) form, prepared from the fast softening ‘AC-F’ and the slow softening ‘AC-S’ genotypes at each firmness category (FC). Data are from ‘season 1’ with one biological replicate which was analyzed using 2-3 technical replicates for neutral sugar and 3 technical replicates for UA analysis. Statistical significance of means between the two genotypes at the same firmness category (FC) is indicated by grey shading (p <0.05). FC, firmness category. Rha, rhamnose; fuc, fucose; ara, arabinose; xyl, xylose; man, mannose; gal, galactose; glc, glucose; UA, uronic acid; FC, firmness category; CWR, cell wall residue

|  | µg mg^-1^ CWM | ‘AC-F’ | ‘AC-S’ |
| --- | --- | --- | --- |
| FC1 | Rha | 6.08 | 1.84 |
|  | Fuc | 0.74 | 0.25 |
|  | Ara | 9.83 | 2.86 |
|  | Xyl | 18.00 | 7.64 |
|  | Man | 4.46 | 2.15 |
|  | Gal | 42.12 | 10.54 |
|  | Glc | 34.69 | 65.78 |
|  | UA | 229.86 | 293.22 |
| FC2 | Rha | 4.46 | 2.96 |
|  | Fuc | 0.70 | 0.37 |
|  | Ara | 7.25 | 4.22 |
|  | Xyl | 21.06 | 10.33 |
|  | Man | 5.82 | 4.33 |
|  | Gal | 33.23 | 13.87 |
|  | Glc | 46.80 | 12.84 |
|  | UA | 216.46 | 295.57 |
| FC3 | Rha | 5.71 | 2.22 |
|  | Fuc | 0.78 | 0.37 |
|  | Ara | 9.29 | 3.08 |
|  | Xyl | 19.48 | 11.24 |
|  | Man | 5.50 | 3.56 |
|  | Gal | 24.43 | 11.00 |
|  | Glc | 16.38 | 9.61 |
|  | UA | 205.43 | 309.59 |
| FC4 | Rha | 3.89 | 1.67 |
|  | Fuc | 0.57 | 0.36 |
|  | Ara | 6.60 | 2.47 |
|  | Xyl | 17.52 | 24.68 |
|  | Man | 4.83 | 5.48 |
|  | Gal | 25.66 | 11.62 |
|  | Glc | 22.63 | 18.54 |
|  | UA | 120.55 | 191.53 |
