# Supplementary material for: Fruit From Two Kiwifruit Genotypes With Contrasting Softening Rates Show Differences in the Xyloglucan and Pectin Domains of the Cell Wall
Source: Front Plant Sci. 2020 Jul 2;11:964. doi: 10.3389/fpls.2020.00964 (PMC7343912; doi:10.3389/fpls.2020.00964)
Supplement: Supplementary file 9 [file Table_6.docx]

**Supplementary Table S6.** Non-cellulosic sugar composition of cell wall extracts prepared from the fast softening ‘AC-F’ and the slow softening ‘AC-S’ genotype of *Actinidia chinensis* var. *chinensis* by sequential extraction of cell wall material over softening. Data are from ‘season 1’ and ‘season 2’, analyzed using at least two technical replicates for neutral sugar and three technical replicates for UA analysis per season. Values are given in µg∙mg^-1^ polysaccharide material in the anhydro (= polymeric) form. Statistical significance of means of monosaccharide residues between the two genotypes at the same FC is represented by grey shading (P <0.05). Rha, rhamnose; fuc, fucose; ara, arabinose; xyl, xylose; man, mannose; gal, galactose; glc, glucose; UA, uronic acid; WS, water soluble; CWR, cell wall residue; FC, firmness category.
