# Supplementary material for: Fruit From Two Kiwifruit Genotypes With Contrasting Softening Rates Show Differences in the Xyloglucan and Pectin Domains of the Cell Wall
Source: Front Plant Sci. 2020 Jul 2;11:964. doi: 10.3389/fpls.2020.00964 (PMC7343912; doi:10.3389/fpls.2020.00964)
Supplement: Supplementary file 10 [file Table_7.docx]

**Supplementary Table S7** Systematic naming of XTH gene models identified in the manually annotated kiwifruit genome (Pilkington et al. 2018) as described in Supplementary Figure S2. Genes were numbered based firstly on established names for kiwifruit XTHs in the literature and then sequentially in cluster order.

| Accession number | Name |
| --- | --- |
| Acc20569.1 | XTH1 |
| Acc00504.1 | XTH2 |
| Acc15005.1 | XTH3 |
| Acc19051.1 | XTH4 |
| Acc14348.1 | XTH5 |
| Acc32677.1 | XTH6 |
| Acc00022.1 | XTH7 |
| Acc08788.1 | XTH8 |
| Acc33618.1 | XTH9 |
| Acc00888.1 | XTH10 |
| Acc18577.1 | XTH11 |
| Acc20468.1 | XTH12 |
| Acc28326.1 | XTH13 |
| Acc17158.1 | XTH14 |
| Acc32179.1 | XTH15 |
| Acc30121.1 | XTH16 |
| Acc32177.1 | XTH17 |
| Acc30122.1 | XTH18 |
| Acc32178.1 | XTH19 |
| Acc33510.1 | XTH20 |
| Acc03173.1 | XTH21 |
| Acc08113.1 | XTH22 |
| Acc09379.1 | XTH23 |
| Acc10242.1 | XTH24 |
| Acc30918.1 | XTH25 |
| Acc05947.1 | XTH26 |
| Acc25038.1 | XTH27 |
| Acc21161.1 | XTH28 |
| Acc09894.1 | XTH29 |
| Acc09780.1 | XTH30 |
| Acc09520.1 | XTH31 |
| Acc13958.1 | XTH32 |
| Acc16608.1 | XTH33 |
| Acc18967.1 | XTH34 |
| Acc00134.1 | XTH35 |
|  |  |
| Acc06234.1 | partial |
| Acc28327.1 | partial |
